# Decoding nitrogen uptake efficiency in maize and sorghum: insights from comparative gene regulatory networks

**DOI:** 10.1111/tpj.70631

**Published:** 2025-12-18

**Authors:** Janeen Braynen, Lifang Zhang, Sunita Kumari, Andrew Olson, Vivek Kumar, Michael Regulski, Christophe Liseron‐Monfils, Allison Gaudinier, Anne‐Maarit Bågman, Shane Abbitt, Mary J. Frank, Bo Shen, Leon Kochian, Siobhan M. Brady, Doreen Ware

**Affiliations:** ^1^ Cold Spring Harbor Laboratory 1 Bungtown Road Cold Spring Harbor New York 11724 USA; ^2^ National Research Council Canada 101 Gymnasium Place Saskatoon Saskatchewan S7N 0W9 Canada; ^3^ Department of Plant and Microbial Biology University of California Berkeley Berkeley California 94720 USA; ^4^ Miller Institute for Basic Research in Science University of California Berkeley Berkeley California 94720 USA; ^5^ Department of Plant Biology and Genome Center University of California Davis Davis California 95616 USA; ^6^ Howard Hughes Medical Institute University of California, Davis Davis California 95616 USA; ^7^ Corteva Agriscience 7300 NW 62nd Avenue Johnston Iowa 50131 USA; ^8^ Department of Plant Sciences University of Saskatchewan Saskatoon Saskatchewan S7N4L8 Canada; ^9^ USDA‐ARS‐NEA Ithaca New York 1485 USA

**Keywords:** nitrogen uptake efficiency, gene regulatory network, transcription factors, nitrate, maize, sorghum, differentially expressed genes, feed‐forward loops

## Abstract

Nitrogen (N) is an essential macronutrient for plant growth and yield, yet optimizing nitrogen use efficiency remains a challenge in agriculture. To better understand the regulatory basis of plant responses to N availability, we constructed a maize‐specific nitrogen uptake efficiency gene regulatory network (mNUEGRN) comprising 1625 protein–DNA interactions (PDI) between 70 promoters and 301 transcription factors using enhanced yeast one‐hybrid assays. We also projected a sorghum NUE GRN (spNUEGRN) based on maize orthologs and analyzed N‐responsive subnetworks in both species using transcriptome profiling under N stress of early deprivation and recovery. Cross‐species comparison with an existing Arabidopsis GRN revealed about 18% conserved interaction, corresponding to 11% of the mNUEGRN, particularly within the nitrate assimilation pathways. Notably, bZIP18 and bZIP30 emerged as central regulators in mNUEGRN, forming highly connected feed‐forward loops (FFLs). From our time series data, we identified 19 236 and 23 864 differentially expressed genes in maize and sorghum, respectively. Gini correlation analysis uncovered 764 and 638 FFLs in mNUEGRN and spNUEGRN, respectively, of which 22 FFLs in maize and 35 in sorghum were identified in both leaf and root for each species. These FFLs may represent candidate regulatory motifs that contribute to modulating transcriptional responses under fluctuating N conditions, but their potential roles require further investigation. Together, our findings reveal evolutionarily conserved and species‐specific regulatory strategies that mediate early N responsiveness, offering a foundation for engineering crops with improved NUE.

## Significance Statement

To decipher the regulatory basis of nitrogen, use efficiency (NUE), we built maize‐ and sorghum‐specific gene regulatory networks (GRNs). Cross‐species analyses revealed conserved and species‐specific feed‐forward loops (FFLs), with bZIP18 and bZIP30 as key regulators in maize. Differential expression and Gini‐based FFL enrichment point to transcriptional buffering under nitrogen stress in both maize and sorghum, offering insights for engineering NUE in crops.

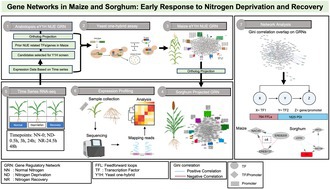

Our study reveals early gene expression responses to nitrogen deprivation and recovery in maize and sorghum. Using a yeast one‐hybrid assay and a derived GRN, we identified transcription factors and nitrogen uptake genes central to early nitrogen response. These regulatory targets provide opportunities for functional validation to improve nitrogen uptake and utilization in staple crops. These findings may have direct implications for reducing agricultural reliance on synthetic fertilizers and advancing sustainable crop production.

## INTRODUCTION

Efficient utilization or uptake of nitrogen (N) is not only a cornerstone for sustainable crop production but also a vital component of responsible environmental stewardship. The overapplication of N fertilizers presents a significant problem; while it boosts crop yields, it simultaneously leads to a host of detrimental environmental impacts. These impacts range from soil and water pollution to a loss of biodiversity and an exacerbation of climate change (Chang et al., 2022; Li et al., 2017). Furthermore, the synthesis of ammonia as N fertilizer from N_2_ has a large carbon footprint, using approximately 1.5% of the world's oil, annually (Woods et al., 2010). Thus, the research community faces the persistent challenge of reconciling the dual goals of reducing fertilizer dependency and maximizing agricultural productivity. Addressing this complex issue requires an in‐depth understanding of nutrient acquisition and use in crops, specifically focusing on the yield of grain, forage, or fruit crop species in direct relation to available N. Achieving higher N acquisition and internal use efficiency is crucial in this regard, as it can lead to optimized functioning and regulation of the key components involved in N assimilation, acquisition, transport/translocation, and signal transduction. While most research has focused on N utilization in *Arabidopsis thaliana*, *Zea mays*, and *Oryza sativa* L. under N deprivation (Chen et al., 2022; Wang et al., 2022), relatively very few studies have explored the early stages of gene expression responses to N via short‐term responses to N deprivation and N recovery (Huang et al., 2025).

As the primary form of N absorbed by crop roots is the NO_3_^−^ anion, which is very mobile in soil and moves with the groundwater, a major aspect of N acquisition from the soil involves the modulation of physiological traits such as root architecture by altering their transcriptome, to maximize nitrate uptake (Ueda, Kiba, & Yanagisawa, 2020; Ueda, Ohtsuki, et al., 2020). To reduce reliance on N fertilizers, researchers and breeders are employing strategies such as genetic engineering, selective breeding, precision agriculture, and systems biology approaches to better understand NUE. Central to these efforts is the understanding of transcriptional regulatory networks, which are far from simple one‐to‐one relationships between genes and transcription factors (TFs). Instead, these networks involve intricate interactions where genes respond to a combination of internal and external signals, orchestrated by a cascade of TFs (Rouached & Rhee, 2017; Ueda, Kiba, & Yanagisawa, 2020; Ueda, Ohtsuki, et al., 2020). These transcriptional networks are regulated by various N acquisition/use‐associated processes, such as root N uptake and transport within the plant, N assimilation, nitrogen‐linked genes, and carbon metabolism to generate amino acids and proteins. Prior studies in *A. thaliana* have utilized systems biology approaches and comprehensive transcriptome analyses to characterize gene regulatory networks (GRNs) and understand responses to different N doses, revealing that key regulators modulate the expression of N‐responsive genes (Brooks et al., 2019; Gaudinier et al., 2018). Additionally, Ueda, Kiba, and Yanagisawa (2020) and Ueda, Ohtsuki, et al. (2020) investigated the transcriptional response of root samples in 20 Asian rice (*O. sativa*) accessions, highlighting specific TFs from the G2‐like and bZIP families as key regulators in response to N deficiency. Members of the bZIP family, in response to N availability, have been shown to be involved in N deficiency stress, along with other TF families such as WRKYs, NODULE INCEPTION‐like proteins (NLPs), Lateral Organ Boundaries Domain (LBDs), and Auxin Signaling F‐box proteins (AFBs) (Singh et al., 2002, 2022; Wu & Chen, 2009). Despite significant research efforts to elucidate complex N transcriptional networks, the majority of these studies have focused primarily on the model plant, Arabidopsis, and mostly the model crop species, rice and wheat, with few studies in maize (Kant et al., 2011; Shanks et al., 2024).

TFs are essential in orchestrating plant development and N response, mediating a range of mechanisms from nutrient sensing to gene regulation. Among these, NLP TFs are crucial for regulating plant development and responding to nitrate availability (Lebedev et al., 2021). Studies have highlighted the role of NLP7 as a nitrate sensor, revealing its time‐dependent mechanisms in Arabidopsis (Liu et al., 2022). This process involves the nitrate‐induced translocation of NLP6 and NLP7 into the nucleus (Cheng et al., 2023; Shanks et al., 2024) and the ‘hit‐and‐run’ model, wherein NLP7 transiently interacts with its target genes. A similar interaction pattern is observed with another TF, bZIP1 (Alvarez et al., 2014; Shanks et al., 2024). Furthermore, NLP7 orchestrates a sequence of downstream TFs, identified through a technique known as ‘network walking’ to uncover how regulatory signals propagate through the TF networks (Brooks et al., 2019). Other TFs that play significant roles in regulating genes for N provision include LBD37‐39, the TGACG motif‐binding factor TFs such as TGA1 and TGA4 (Swift et al., 2020). Additionally, ARABIDOPSIS NITRATE REGULATED 1 (ANR1) and AUXIN RESPONSIVE FACTORs (ARFs), have been identified as integral components involved in root development through nutrient sensing control (Crawford & Glass, 1998; Vidal et al., 2013; Zhang & Forde, 1998). Moreover, Gaudinier et al. (2018) used an enhanced yeast one‐hybrid (Y1H) approach to construct a nitrogen‐associated regulatory network in *A. thaliana*, revealing that mutations in 17 TFs significantly altered root system architecture (RSA) under different nitrogen regimes. These included well‐characterized regulators such as NLP7, GNC, and ARF9/ARF18 along with additional TFs like DREB26 and ANACO32, emphasizing the extensive overlap between nitrogen signaling and RSA control (Gaudinier et al., 2018). Consequently, a nuanced understanding of the dynamics between nitrate availability and its genetic regulation is pivotal for optimizing plant nitrogen utilization in varied nitrate environments.

To ensure plants effectively utilize N, a critical macronutrient, they deploy a range of transport mechanisms. These transporters primarily acquire nitrogen in the forms of nitrate (NO_3_^−^) and to a lesser extent, ammonium (NH_4_^+^), while crop roots also have the ability to absorb the N fertilizer, urea (CO(NH_2_)_2_), which is converted biochemically in the plant into the synthesis of amino acids and then proteins (Glass, 2003; Kojima et al., 2007). This conversion into amino acids involves complex molecular pathways (Coruzzi & Zhou, 2001; Forde & Lea, 2007). Specific transporters enable plants to acquire from the soil, accommodating both inorganic (nitrate and ammonium ions) and organic compounds such as urea (Tegeder & Masclaux‐Daubresse, 2018). Integral to this system are diverse transporter families, such as the nitrate transporters (NRTs) and ammonium transporters (AMTs) (Léran et al., 2014; von Wirén et al., 2000). A specific NRT, NRT1.1 also functions as a nitrate transceptor that acts both as a transporter and receptor (Bouguyon et al., 2012). Furthermore, NRT1.1 also functions as an auxin transporter, which is involved in nitrate‐mediated changes in root architecture (Bouguyon et al., 2015). As plants predominantly rely on these transporters for nutrient uptake, extensive research has been dedicated to understanding the exact functions of nitrate transporter genes. In maize, for instance, ZmCHB101 modulates nitrate‐responsive transporters like *ZmNRT2.1* and *ZmNRT2.2*, promoting root growth by facilitating auxin transport in N‐limiting scenarios (Meng et al., 2020). An earlier study reported by Okamoto et al. (2006) on N deprivation in *A. thaliana* highlighted the significant roles of *AtNRT3.1* and *AtNRT3.2* genes in nitrogen transport. Environmental N availability has also been linked with the regulation of nitrate transporter genes when other nutrients are deficient. Therefore, understanding the expression and regulation patterns of specific N uptake and utilization mechanisms across various plant species may clarify the regulatory relationships among them.

One of the primary challenges in studying NUE is unraveling the complex interplay of underlying processes, particularly the transcriptional regulation mechanisms responding to N availability. To address this, we first leveraged our previous work on the Arabidopsis N‐associated metabolic GRN to identify orthologous genes and conserved transcriptional interactions in maize. Using a Y1H assay, we built an enhanced maize Y1H network (eY1H) (Gaudinier et al., 2018). This network was then overlaid with global spatiotemporal transcriptome data from N deprivation and recovery experiments and applied Gini‐correlation analysis (Liseron‐Monfils et al., 2018). We further compared this to a projected sorghum network to analyze conserved responses in maize and sorghum. This integrative approach enabled the identification of conserved protein–DNA interactions (PDI) among TFs implicated in N metabolism. Our findings highlight dynamic regulatory networks in cereal crops and emphasize the conservation of N‐responsive mechanisms between monocots and dicots.

## RESULTS

### Dissecting the maize GRN for key TFs and nitrogen uptake modules

**Figure 1:**
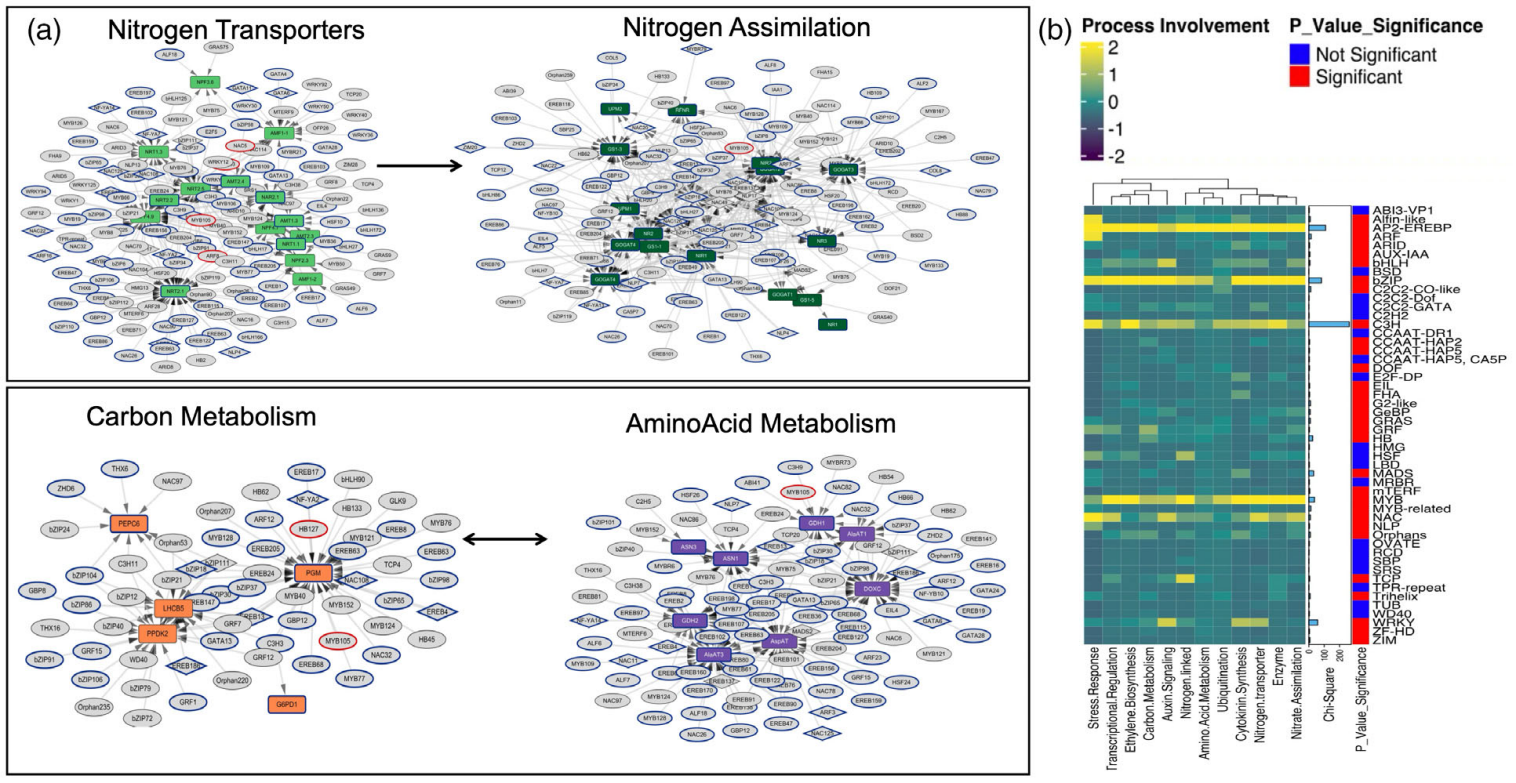
Overview of maize gene regulatory network statistics. (a) Nitrogen acquisition (nitrogen transporters; nitrogen assimilation) and C–N metabolism (carbon metabolism; amino‐acid metabolism) network from maize nitrogen uptake efficiency gene regulatory network. Colored rectangles mark curated pathway genes. Rectangles indicate promoters, ovals represent transcription factors (TFs), and diamonds symbolize genes that function as both promoters and TFs. (b) The heatmap delineates the activity of TF families across these biological processes, where color intensity correlates with activity level, vertical bars represent chi‐square statistics for each TF family, and significance is marked by red (*P* < 0.05) for significant and blue for nonsignificant *P*‐values. Various shapes and colors denote different components and processes the network.

In our study, we employed eY1H assays to identify TFs that regulate N metabolism in maize (Table S1a,b). The genes screened via eY1H in maize were based on orthologous projections from the Arabidopsis eY1H GRN elucidated in Gaudinier et al. (2018) (Figure S1). To dissect the complexity of N metabolism regulation, we selected target promoters for genes involved in a wide array of N‐related processes: N transport, N assimilation, amino acid and carbon metabolism, organ growth and hormone responses (Figure 1a; Table S2a). Additionally, we expanded the muYIHGRN TF collection to include key regulators such as DREB2A, ANR1, NAC1, NAC032, and AthWRKY18/40/60, which are central TFs in the Arabidopsis regulatory network. Furthermore, we have expanded the maize GRN by incorporating promoters for TFs to explore the hierarchical interactions among TFs. The eY1H maize network comprises 301 TFs from 42 TF families and 70 promoters, forming 1625 PDIs (Figure S2a). The distribution of indegree (the number of TFs that bind to a promoter) and outdegree (the number of promoters to which a TF binds) is shown in Figure S2b, and reflects patterns commonly observed in other biological networks, with a few highly connective hubs and many nodes with few connections (Table S2b). On average, each promoter within the GRN modules has an indegree of 10 or more interactions from TFs; however, 28 promoters have 5 or fewer TF interactions (Figure S2b; Table S2c). Notably, 16 promoters within GRN modules are bound by more than 35 TFs. These highly connected promoters include the high‐affinity N transporter NRT2.1, the low‐affinity N transporter NPF4.9, the enzymes AlaAT, Glutamine synthetase genes GS1‐3 and GOGAT2, nitrate reductase NIR1, Phosphoglucomutase (PGM), the auxin signaling component TIR1, and the NLP6 (ortholog of AtNLP6), and NLP17 (ortholog of AtNLP7) TFs (Table 1). Collectively, these genes represent central components of nitrogen uptake, assimilation, and regulatory signaling. Among the TFs identified, 16 TFs bind to over 20 promoters. Eleven of these 16 TFs belong to the AP2‐EREB, bZIP, and MYB families. Furthermore, 212 TFs were found to bind to five or fewer promoters, emphasizing the ability of the eY1H screen to capture the binding specificity of individual TFs. To determine whether specific TF families exhibited significantly higher regulatory connectivity than expected by chance, we performed a chi‐square goodness‐of‐fit test comparing the observed outdegree. The total network comprises 1625 outgoing edges among all TFs, so the expected outdegree of a given TF family was calculated by considering its proportion of the total TFs (301 in the network) (Figure S3; Table S3a). From this test, the distribution of TFs indicated that C3H (*P* = 3.96E‐59), AP2‐EREBP (*P* = 1.24E‐25), bZIP (*P* = 1.15E‐19), WRKY (*P* = 2.65E‐14), and MYB (*P* = 1.22E‐09) were indeed significantly overrepresented within the network (Figure 1b; Table S3b,c). While these results support the central regulatory roles of these TF families, we acknowledge that high connectivity in eY1H data can sometimes reflect TFs with broad DNA‐binding properties. We therefore interpret these highly connected TFs as strong candidates for hub regulators in nitrogen‐associated transcriptional networks and can be used for the experimental validation for future research.

**Table 1:** Highly connected promoters in the maize‐specific nitrogen uptake efficiency gene regulatory network (mNUEGRN)

Gene ID	Gene name	Indegree	Outdegree	Node type*	Ath gene name	Process
Zm00001eb061150	DUB	36	0	P	AT2G39210	Ubiquitination
Zm00001eb329780	HAD2	36	0	P	AT1G14310	Enzyme
Zm00001eb410960	DOXC	36	0	P	AT1G03400	Amino acid metabolism
Zm00001eb193660	NIR1	36	0	P	AthNIR1	Nitrate assimilation
Zm00001eb093650	NAC126	37	4	T/P	AthCUC2	Transcriptional regulation
Zm00001eb253820	GS1‐3	37	0	P	AthGLN1;2	Nitrate assimilation
Zm00001eb118950	NLP6	38	0	P	AthNLP6	Transcriptional regulation
Zm00001eb361110	NRT2.5	39	0	P	AthNRT2.2	Nitrogen transporter
Zm00001eb104260	NLP17	39	4	T/P	AthNLP4	Transcriptional regulation
Zm00001eb068280	AspAT	40	0	P	AthASPGB1	Amino acid metabolism
Zm00001eb327530	AlaAT3	40	0	P	AthALAAT2	Amino acid metabolism
Zm00001eb194260	PGM	42	0	P	AThPGM1	Carbon metabolism
Zm00001eb078860	NPF4.9	43	0	P	AT1G27040	Nitrogen transporter
Zm00001eb360480	GOGAT2	44	0	P	AthGLT1	Nitrate assimilation
Zm00001eb113030	TIR1	47	0	P	AthAFB2	Auxin signaling
Zm00001eb209670	NRT2.1	55	0	P	AthNRT2.1	Nitrogen transporter

*Note: Node type—There are two types of nodes: P: promoter, T/P: TF and promoter.

Out of 301 TFs belonging to 42 TF families within the maize GRN, 54% (163TFs) from the AP2‐EREBP, bZIP, NAC, and MYB TF families collectively account for 63% of all promoter–TF interactions in the network. The AP2‐EREBP family, which contains 53 members (18% of all TFs), contributes to 28% of all outgoing interactions (447/1625 edges) (Figure S3; Table S4a–c). The bZIP family comprises 30 members (10% of all TFs), accounts for 17% of the outgoing edges (270/1625 edges) with individual members such as ZmBZIP30 and ZmbZIP18 binding to 2.5% (42/1625) and 3% (48/1625) of all promoters, respectively (Figure S3; Table S4d,e). The MYB family, slightly smaller than AP2/EREBP and bZIP, demonstrates significant activity with 13.6% (217/1625) (Table S4f). To further study the network, we analyzed the overrepresented TF families within each functional submodule which was highly targeted. Given the more frequent presence of TF families within the network, and their overrepresentation of critical N‐associated metabolic genes, we hypothesized that these represent functional modules associated with discrete metabolic activities.

**Figure 2:**
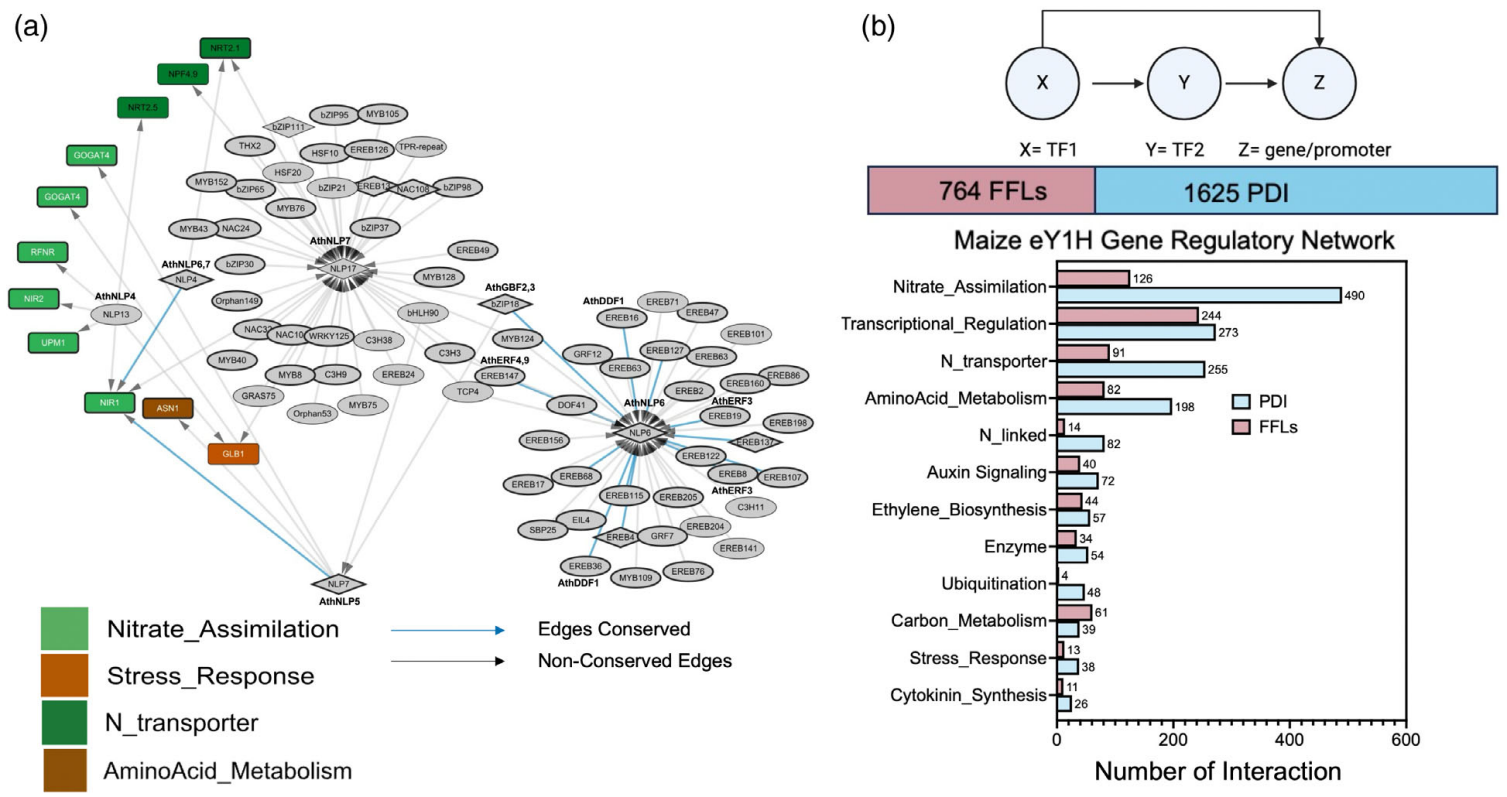
Conserved interactions between Arabidopsis and maize. (a) Submodules of the NODULE‐INCEPTION‐like proteins (NLPs) family member in the maize gene regulatory network (GRN). The light blue lines are the conserved edges between Arabidopsis and maize submodules of the NLP family. (b) Schematic diagram of feed‐forward loops and the statistics of those in the GRN for maize with pink representing the number of feed‐forward loops, and blue representing the total number of PDI interactions in the network.

Furthermore, our assays confirmed previously characterized interactions of the Arabidopsis NLP family in maize that regulate key genes involved in N metabolism, with some new interactions and additional conserved interactions when compared to maize (Figure 2a). In maize, we introduced ZmNLP17, ZmNLP7, ZmNLP4, and ZmNLP13 into the regulatory network, observing 79 indegrees and 18 outdegrees for these TFs (Figure 2a). ZmNLP4, a maize ortholog of AthNLP6, displayed a conserved interaction with the nitrate reductase (NIR1) promoter, consistent with AthNLP6–NIR1 interactions in Arabidopsis. By contrast, ZmNLP17 (ortholog of AthNLP7), also interacted with NIR1 but this interaction was not conserved when comparing AthNLP6–NIR1 interactions. Moreover, within the maize eY1H GRN, we identified 764 potential interactions between TF families forming ‘three nodes’ motifs indicating TF1 binds to TF2 which both bind to a common promoter (Figure 2b; Table S5). We observed that the proportional representation of TF families within the network is reflected in their occurrence in these motifs. The distinct regulatory roles of these TFs alongside the identification of these motifs within the maize‐specific nitrogen uptake efficiency (NUE) GRN (mNUEGRN), suggest a complex network architecture that governs N metabolism.

In dissecting the maize GRN, four key functional modules (N assimilation, N transporter, carbon metabolism, and amino acid metabolism) were analyzed. The N assimilation module contains genes predicted to be critical for converting inorganic N into organic forms usable by the plant, was heavily populated with promoters bound by TFs from the AP2/EREBP family (85 interactions), the MYB family (49 interactions), and the bZIP family (37 interactions). Within the N transport module, the AP2/EREBP family exhibits extensive involvement with 47 interactions, followed by the bZIP family with 34 interactions. The amino acid metabolic module contains key enzymes involved in synthesizing essential amino acids. Here, the AP2/EREBP family had over 102 interactions with these enzyme promoters, and the bZIP family had 23 interactions. The carbon metabolic module contains enzymes interlinked with N metabolism which could coordinate the provision of the carbon backbones for amino acid metabolism. In this module, the bZIP family demonstrates the highest connectivity with 33 interactions. These discrete modules likely ensure a balanced interplay between carbon and N assimilation processes via regulation by different TF families.

### Temporal dynamics of gene expression and network analysis in response to N deprivation and recovery in maize

To better understand the maize GRN, we investigated global gene expression in response to N starvation and recovery. Our research employed a two‐phase experimental design in hydroponics to capture the transcriptomic responses and regulatory networks associated with N depletion and recovery. The N depletion phase transitioned from normal N to low N with samples collected at 0.5, 3, and 24 h, followed by a recovery phase from low N to normal N with samples collected at 24.5 and 48 h. The early time point 0.5 h was selected to capture immediate transcriptional responses, including the primary response genes and early signaling cascades. Previous studies (Dechorgnat et al., 2019; Wang et al., 2000) have shown that nitrogen responsive TFs and signaling components can be activated within 30 min to 1 h of nitrogen treatment. A 3‐h time point was selected as an intermediate time point to capture secondary and tertiary responses as downstream regulatory networks are activated. Finally, the 24‐h time point was used as the extended time point to capture long‐term adaptive adjustments of the transcriptome to either nitrogen limitation or recovery. The rationale for implementing nitrogen recovery after 0.5 and 24 h of N limitation (24.5, 48 h) was to provide a comprehensive temporal framework to track the full trajectory of plant transcriptional responses, from initial sensing through acclimation and adaptation. While this design does not necessarily replicate the longer term, variable N deprivation encountered in field conditions, it provides a controlled framework for probing the initial stages of N signaling. Furthermore, because all forms of N fertilizer ultimately are converted primarily to NO_3_^−^ by soil microbial activity, and nitrate is the most mobile fertilizer ion in soils, moving with the groundwater, there can be significant spatial variability in soil nitrate concentrations. Hence, roots, even in well‐fertilized soils, can grow into low nitrate soil regions and thus encounter localized soil N deprivation.

**Figure 3:**
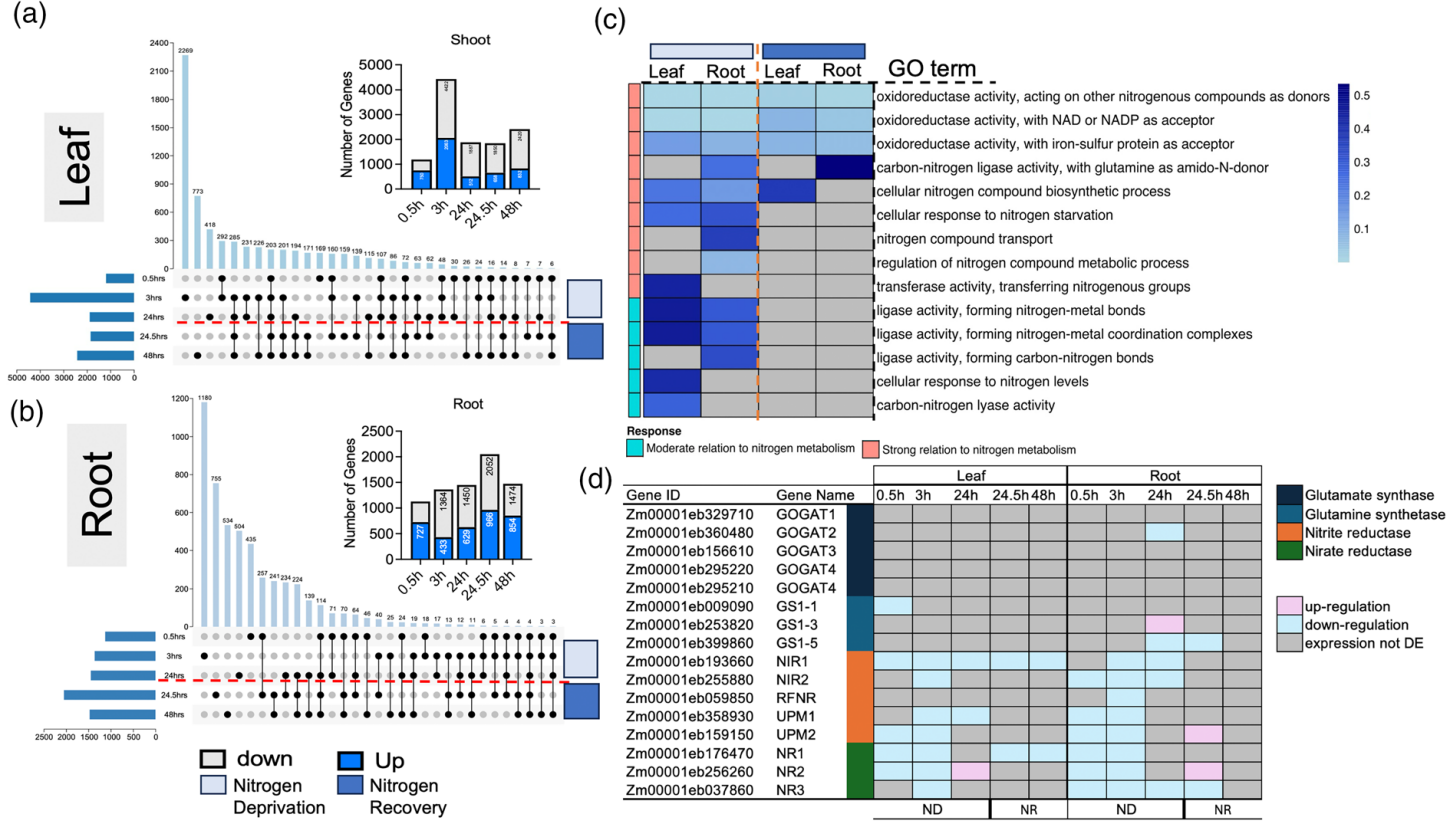
Spatiotemporal transcriptomic dynamics in maize under varying N conditions in leaf and root. (a, b) UpSet plots illustrate the number of differentially expressed genes with distinct temporal expression patterns under 0.5, 3, and 24 h under limiting nitrogen and at 24.5 and 48 h time points after recovery in leaves and roots. (c) Gene Ontology analysis of nitrogen‐related ontology in leaf and roots which are overrepresented. (d) Differentially expressed genes in the nitrogen assimilation modules (upregulation is depicted in pink and downregulation blue). ND, nitrogen deprivation; NR, nitrogen recovery.

Expression analysis within leaf and root tissues in maize revealed genes with differences in N‐responsiveness specific to time points and/or tissue analyzed. We observed dynamic transcriptional changes in both leaves and roots in maize across all the time points of nitrogen deprivation and recovery. Differential expression analysis in maize identified a total of 11 768 genes in leaves and 7468 in roots across all time points (Tables S6 and S7). There were a total of 7498 genes in leaves and 3942 in roots differentially expressed during the N deprivation. For N deprivation, early responses in the leaves were 10% (1189/11 768 genes) affected at the 0.5‐h time point (Figure 3a). The strongest response occurred at the 3‐h time point, where 38% (4422/11 768 genes) were differentially expressed in leaves. This was followed by 16% of differentially expressed (1887/11 768 genes) at the 24‐h time point. In contrast, root tissues showed a gradual increase in the number of differentially expressed genes over time. At early deprivation time points (0.5 and 3 h), 15% (1128/7468 genes) and 18% (1364/7468 genes) were differentially expressed, respectively. Interestingly, by 24 h, roots displayed a slight increase in expression changes 19% (1450/7468 genes) (Figure 3b; Table S7). Functional enrichment analysis of the differentially expressed genes during nitrogen deprivation revealed significant enrichment in pathways related to oxidoreductase activities (likely nitrate and nitrite reductase activities) and known nitrogen starvation responses in both leaf and root tissues (Figure 3c). Among these, oxidoreductase activity acting on nitrogenous compounds as donors showed the strongest enrichment in both leaves (*P* = 0.00214) and roots (*P* = 9.4 × 10^−6^) during nitrogen deprivation. However, although pathways associated with cellular responses to nitrogen starvation showed enrichment, these trends were not statistically significant in either leaves or roots. In contrast, N transport was specifically enriched in roots, supporting the observation that genes involved in root N transport processes were among the earliest to respond to nitrogen deprivation. Furthermore, NAD/NADP‐dependent oxidoreductase activity was significantly enriched in roots (*P* = 2.56 × 10^−5^) (Table S8). Together, these results highlight a strong and tissue‐specific transcriptional reprogramming during nitrogen deprivation, with roots exhibiting distinct enrichment of transport‐ and redox‐related processes presumably associated with nitrate and nitrite reduction.

Following nitrogen resupply (24.5–48 h), distinct recovery‐associated transcriptional responses emerged. There were a total of 4270 genes in leaves and 3526 in roots differentially expressed during the recovery phase. In leaves, 16% (1850/11 768 genes) were differentially expressed at 24.5 h, similar in magnitude to the deprivation response at 24 h, while 21% (2420/11 768 genes) were affected at 48 h of the transcriptome (Figure 3a). In roots, the largest transcriptional shift 27% (2052/7468 genes) occurred during the early recovery phase at 24.5 h. At 48 h, the response stabilized slightly, with 20% (1474/7468 genes) differentially expressed (Figure 3b). These trends highlight the temporal and tissue‐specific dynamics of nitrogen recovery, with leaves showing persistent adjustments, while roots displayed a pronounced but transient surge in gene expression upon resupply. Enrichment analyses during recovery indicated continued involvement of nitrogen‐related processes, particularly oxidoreductase activities in both tissues. However, in contrast to the deprivation phase, none of these enrichments reached statistical significance. To further investigate these patterns, we integrated the expression data with the mNUEGRN to explore shifts in regulatory activity associated with nitrogen deprivation and subsequent recovery.

We wanted to explore mNUEGRN using spatio‐temporal transcriptomic responses obtained from the differential gene expression data in both leaves and roots. In leaves, 31, 84, and 32 nodes in the mNUEGRN were differentially expressed at 0.5, 3, and 24 h, respectively, representing early response phases. In roots, 54, 18, and 46 nodes were differentially expressed at the same time points. During recovery, 39 nodes were differentially expressed in leaves at both 24.5 and 48 h, while in roots, 66 and 30 nodes showed differential expression at these time points, respectively. For genes associated with primary and secondary metabolic pathways, regulation in response to N stress appeared more tissue‐specific across modules (Table S9a). For instance, the genes LHCB5, PDK2, PGM, and PEP4 involved in C metabolism were predominantly downregulated in leaves during various N deprivation time points, whereas in roots, ZmG6PD1 and PGM were downregulated at 3 h, and LHCB5 was upregulated at 48 h. Interestingly, among plant hormone signaling pathway genes, LOG2, related to cytokinin biosynthesis, was downregulated at 3, 24.5, and 48 h in leaves but did not exhibit differential expression in roots. Additionally, the auxin signaling gene, ARF4, was upregulated exclusively in roots at 0.5 and 24 h, transitioning to N recovery at 24.5 h.

Our analysis indicated that, although few genes in the GRN responded to early N deprivation, two major modules, N assimilation and N transport, were significantly affected by N availability. Within the N assimilation pathway, numerous genes showed time‐specific up‐ and downregulation, with more pronounced changes in roots compared to leaves (Figure 3d). Notably, early response genes such as NIR1, NR3, NR1, NIR2, and UMP1 were consistently downregulated. In leaves, nitrite reductase NIR1 was downregulated across all time points, whereas in roots, it was downregulated only at 3 and 24 h. Nitrate reductase NR3 was downregulated at all time points except 48 h, and NR1 was downregulated during early time points in both tissues; however, in leaves, NR1 remained downregulated upon N recovery. These findings indicate that N assimilation‐related genes respond early to N deprivation, with distinct regulation patterns in leaves and roots. For N transporters, we observed four genes showing consistent regulatory patterns in roots and leaves to N reduced availability. Among high‐affinity transporters, the ammonium transporter, AMT1.3, was consistently downregulated at all time points in leaves and at 24 and 24.5 h in roots, while the nitrate transporter, NRT2, was predominantly upregulated in roots at all time points except 48 h. Among low‐affinity transporters, NPF3.6 was mainly downregulated in leaves, and NRT1 showed a similar pattern to NRT2, being primarily upregulated in roots at all time points except 48 h. Together, these results indicate that N transporters respond rapidly and, in a tissue‐specific manner to N deprivation in maize, with decreased expression of AMTs and concurrent induction of nitrate transporters, particularly in roots.

To further elucidate regulatory dynamics under N conditions, we identified correlated expression patterns between TFs and target genes using the NECorr package (Liseron‐Monfils et al., 2018), which employs the Gini correlation coefficient. Our network analysis revealed 167 negative interactions in leaves and 160 in roots, alongside 278 and 238 positive interactions in leaves and roots, respectively (Table S10; Figure S4a). These correlations highlighted specific modules within the mNUEGRN that were strongly associated with N response. When using Gini correlation, we observed a positive Gini correlation between C metabolism‐associated genes LHCB5, PDK2, and TFs ZmbZIP12 and ZmC3H11, with both genes; these TFs (ZmbZIP12 and ZmC3H11) showing downregulation under N deprivation and recovery (Figure S4; Table S9b). For PGM, an enzyme that plays a key role in carbohydrate metabolism, most interactions were positively correlated, while phosphoenolpyruvate carboxylase 4 (PEP4) exhibited a single positive correlation with ZmbZIP18, although this TF was not differentially expressed in our maize study. In roots, PGM was associated with eight TFs with positive correlations and three with negative correlations, including ZmOrphan207 (a grass‐specific TF), ZmNAC108, and HB62. Additionally, ZmbZIP18 demonstrated a positive correlation with pyruvate, orthophosphate dikinase2 (PDK2) in roots, though PDK2 was under N deprivation and recovery in maize.

**Figure 4:**
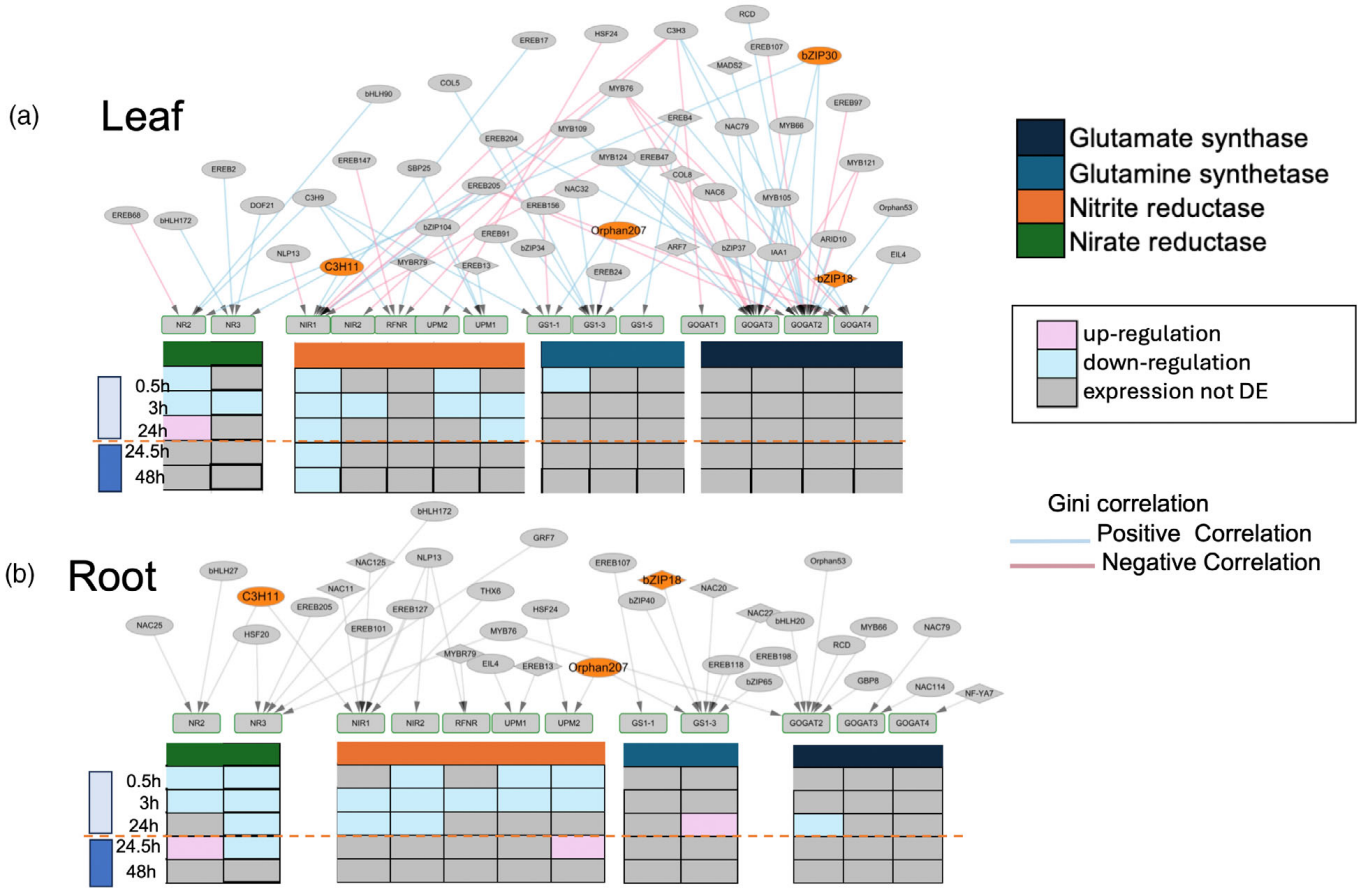
Transcriptomic analysis and Gini correlation in the N assimilation module of the maize yeast one hybrid gene regulatory network (mNUEGRN). (a) Expression patterns of N assimilation genes in leaves under N deprivation and recovery, with Gini correlations mapped onto the module. (b) Expression patterns of N assimilation genes in roots under N deprivation and recovery, with Gini correlations similarly mapped. Upregulated genes are indicated in pink, while downregulated genes are shown in blue. Positive Gini correlations are represented in blue, and negative interactions are shown in pink.

Within N assimilation pathways in leaves, TFs displayed specific targeting patterns, with GOGAT2/3 showing positive correlations with members of the bZIP family (e.g., ZmbZIP18 and ZmbZIP30) as well as MYB family members (Figure 4a). NIR1 was positively correlated with C3H11, and GS1‐3 was positively correlated with ZmOrphan207 and bZIP34, along with several other TFs. In roots, GOGAT1 displayed a positive correlation with C3H11, while GOGAT2 exhibited both positive and negative correlations, suggesting condition‐dependent regulation. Notably, GS1‐3 was targeted by three bZIP TFs: ZmbZIP65 (positive), ZmbZIP40 (negative), and ZmbZIP18 (negative), as well as ZmOrphan207 with a positive correlation (Figure 4b). For nitrate and nitrite reductases, NR2 and NIR1 were positively regulated by C3H11. Additionally, for N transporters, ZmNPF4.9 in leaves was targeted by 16 TFs, including ZmbZIP30 and NLP17, while roots displayed fewer correlated interactions (Figure S5). Nonetheless, NRT2.1 in roots was positively regulated by ZmOrphan207. Additionally, ZmbZIP11, a common target of ZmbZIP18 and bZIP30, exhibited a positive correlation, suggesting coordinated regulation in response to N availability. These regulatory relationships emphasize complex TF‐mediated control over N response genes, pointing to tissue‐specific and context‐dependent activation and repression patterns.

**Figure 5:**
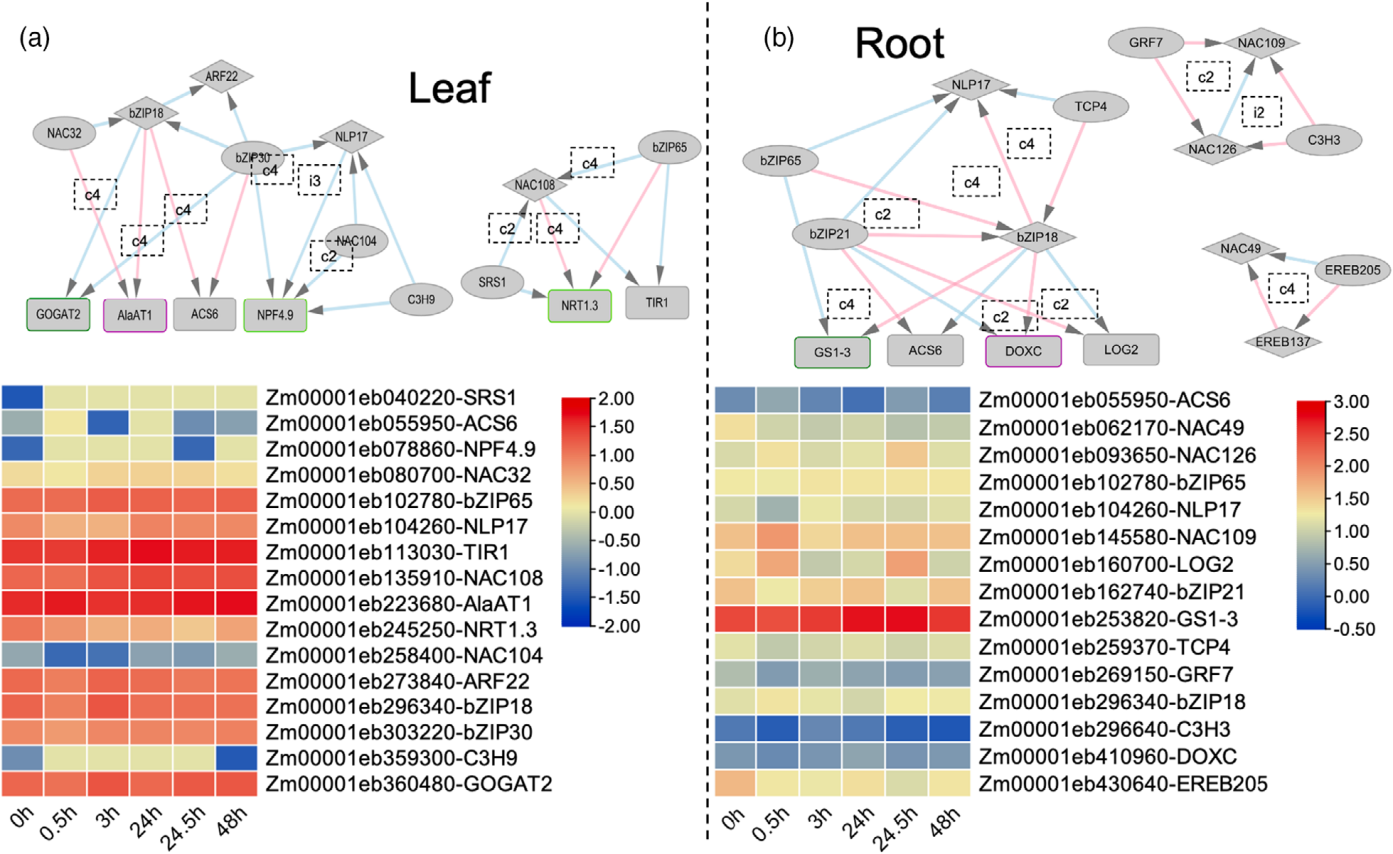
Feed‐forward loops (FFLs) in the transcriptional response to nitrogen stress. (a) Significant FFL interactions in leaf tissue within the maize‐specific nitrogen uptake efficiency gene regulatory network (mNUEGRN) network, illustrating regulatory motifs that shape nitrogen‐responsive gene expression. Heatmaps display gene expression patterns across various time points under N deprivation and recovery, with rows representing target genes and columns representing time intervals. (b) Significant FFL interactions in root tissue, similarly, highlighting regulatory motifs within the mNUEGRN that govern nitrogen response phases. Coherent FFL types (c1–c4) and incoherent FFL types (i1–i4) indicate consistent versus dynamic regulatory strategies, respectively, emphasizing tissue‐specific adaptations to N stress. The borders around the promoters depict which module that promoter was a part of. Purple: auxin signaling, dark green: nitrate assimilation and light green: nitrogen transporters.

Based on the Gini correlation data, we aimed to explore potential feed‐forward loop (FFL) motifs in the mNUEGRN due to the hierarchical organization of TFs within the network. Our analysis identified approximately 764 potential FFL interactions in the mNUEGRN. However, we emphasize that while FFL motifs can be inferred computationally, the current dataset does not allow for disentangling the independent regulatory contributions of each TF within these loops. Consequently, the categorization of FFLs into coherent or incoherent subtypes presented here is to enable the generation of additional hypotheses about the interaction patterns and their effects on the network. Using this exploratory approach, we identified 11 candidate FFLs in leaf tissues and 12 in roots. In leaves, several FFLs involve TFs such as C3H9, bZIP30, NAC104, and SRS1, each associated with N‐responsive gene regulation (Figure 5a). For example, C3H9, bZIP30, and NAC104 form potential FFLs with NLP17, targeting NPF4.9, a key N transporter. These include patterns resembling coherent Type 2 (c2), coherent Type 4 (c4), and incoherent Type 3 (i3) configurations, suggesting diverse regulatory strategies that may integrate both activating and repressive inputs. Additional candidate motifs involve NAC108 with SRS1 and bZIP65 targeting *NRT1.3*. Interestingly, NAC108 has been previously implicated in regulating leaf senescence and photosynthetic apparatus degradation (Podzimska‐Sroka et al., 2015; Zhang et al., 2019), consistent with its placement in a leaf FFL that converges on auxin signaling (*TIR1*), potentially linking senescence and hormone‐mediated responses to N signaling. Other candidate motifs include bZIP18 with NAC32 and bZIP30 co‐targeting genes such as *AlaAT1*, *ARF22*, and *GOGAT2*, indicating putative mechanisms to coordinate N assimilation. In roots, FFLs featuring bZIP21 and bZIP18 exhibit patterns consistent with coherent Type 2 (c2) motifs targeting *NLP17*, *LOG2*, and *ACS6*, potentially stabilizing gene expression under N deprivation (Figure 5b). Similarly, bZIP18 appears in putative coherent Type 4 (c4) FFLs with TCP4 and bZIP65 targeting *GS1‐3*, an enzyme critical for N assimilation. In contrast, patterns resembling incoherent Type 2 (i2) FFLs, such as C3H3 and NAC126 targeting NAC109, could support flexible regulation under fluctuating N conditions. We also detected an FFL involving EREB205 and EREB137 that converges on NAC49, consistent with a possible reinforcing mechanism for NAC49 expression. While these predicted motifs provide a framework for future work incorporating N perturbation experiments and temporal expression profiling, which will be essential to validate the roles of these candidate FFLs in N‐responsive regulation.

### Comparative analysis of protein–DNA interactions in Arabidopsis and maize NUE GRNs

To identify conserved regulatory interactions in N assimilation, we identified conserved PDIs using ortholog/homolog predictions between the maize network mNUEGRN and the previously published Arabidopsis network (aNUEGRN) by Gaudinier et al. (2018) (Figure S6a). Our methodology for determining conservation was stringent; an interaction was considered conserved only if it involved orthologous genes (both promoter and TF) that were experimentally validated in both Arabidopsis and maize. Conversely, interactions that featured just one orthologous component, either a promoter or a TF, were not classified as conserved. This criterion allowed us to distinguish interactions that are shared across species (conserved), from those detected only in maize, thereby highlighting putative species‐specific regulatory connections. The projection of aNUEGRN to maize resulted in a merged network comprising 26 014 PDIs involving 226 promoters and 1165 TFs. To measure the degree of conservation, we specifically focused on interactions tested in the maize and Arabidopsis Y1H screens (Table S11a,b). The Arabidopsis Y1H network includes 603 edges that correspond to 1529 edges in the maize eY1H screen. Our quantitative analysis indicated that 18% (111/603) of the tested interactions in Arabidopsis were conserved compared to 11% (162/1529) of the maize GRN (Table S11a,b). The conservation of N assimilation and N‐linked genes was notably high between Arabidopsis and maize (Figure S6a). Specifically, the N assimilation pathway showed 38 conserved edges in Arabidopsis and 65 in maize (Figure S6a,b), with higher conservation in maize, reflecting its genome duplication events. Key conserved interactions were identified across both species, including orthologous relationships such as AthMYB9 and ZmMYB105, which bind to AthGLT1/ZmGOGAT, and the likely regulatory role of AthERF8 on AthGLT1/ZmEREB147, both targeting the AthGLT1/ZmGOGATA promoter (Table S11c,d). We further examined N transporter interactions between Arabidopsis and maize. In maize, we observed that 12% (191/1529) of edges were associated with N transporters. Among these, 5.2% (10/191) of interactions showed conservation to 10.3% (9/87) of the N transporters identified in Arabidopsis (Table S11c,d). In Arabidopsis, TFs such as AthERF4, AthERF9, AthGBF2, AthGBF3, and AthSFB2A target the promoter of AthNRT3.1, whereas in maize, ZmNRT3.1 is similarly targeted by ZmEREB147 and ZmHSF10 and by ZmbZIP18. Additionally, both species exhibit conservation of the ARF family, with AthARF4 binding to NRT2.4, and its maize orthologs ZmARF28 and ZmARF8 targeting the high‐affinity nitrate transporter ZmNRT2.4. ZmEREB147 in maize could play a key regulatory role, targeting a diverse set of N response‐related genes, including maize orthologs of AthNPF6.3 (ZmNPF1) and AthNPF2, as well as AT1G61730 and ZmGBP8, both of which interact with ZmNRT2.1. These findings position ZmEREB147 and ZmbZIP18 as pivotal and conserved TFs in maize, underscoring a complex, species‐specific regulatory architecture within maize N metabolism networks. Possibly, ZmEREB147 is modulating ZmNLP6, an ortholog of AtNLP7, a known regulator in N metabolism. Unlike EREB family members, bZIP TFs, particularly ZmbZIP18 and ZmbZIP30 (orthologs of AtGBF2 and AthGRF1), demonstrate broader interaction profiles, with ZmbZIP18 notably integrating hormone‐specific pathways and regulating ethylene‐related enzymes such as ZmACS1/2/6/7 (Figure S7).

### Comparative analysis of GRNs in maize and sorghum reveals complex nitrogen‐responsive dynamics

**Figure 6:**
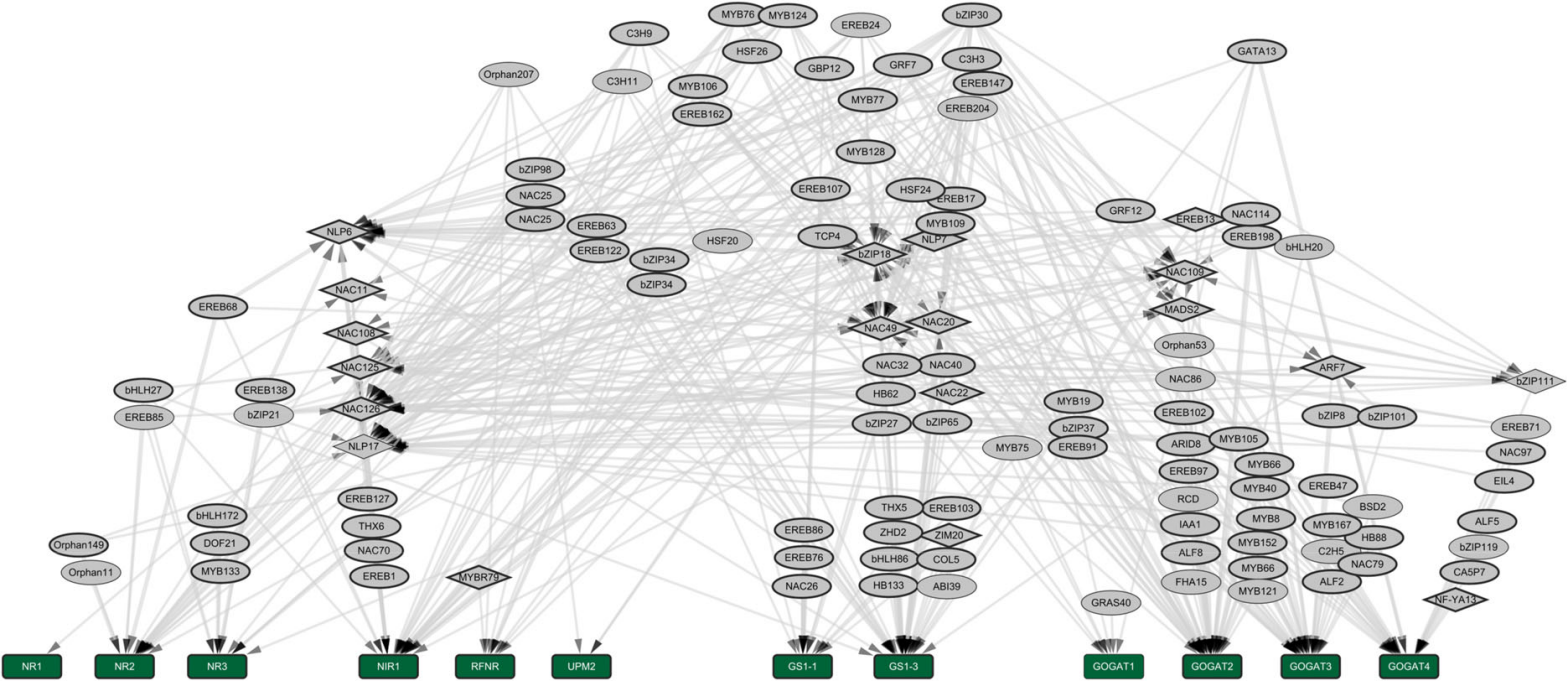
Transcriptional regulatory network of nitrogen assimilation genes. Network visualization of transcription factors (TFs, gray nodes) predicted to regulate key genes in the nitrogen assimilation pathway (green nodes). Nitrogen reductase genes (NR1, NR2, and NR3), nitrite reductase (NIR1), glutamine synthetases (GS1‐1 and GS1‐3), glutamate synthases (GOGAT1, GOGAT2, GOGAT3, and GOGAT4), and the uroporphyrinogen methyltransferase (UPM2) are shown as central target nodes. Directed edges represent regulatory interactions predicted from gene expression data and/or motif enrichment analysis, with arrowheads pointing toward the putative target gene. Node size is proportional to connectivity (number of predicted interactions), and edge thickness reflects interaction confidence. For the entire projected sorghum gene regulatory network, see Figure S8.

**Figure 7:**
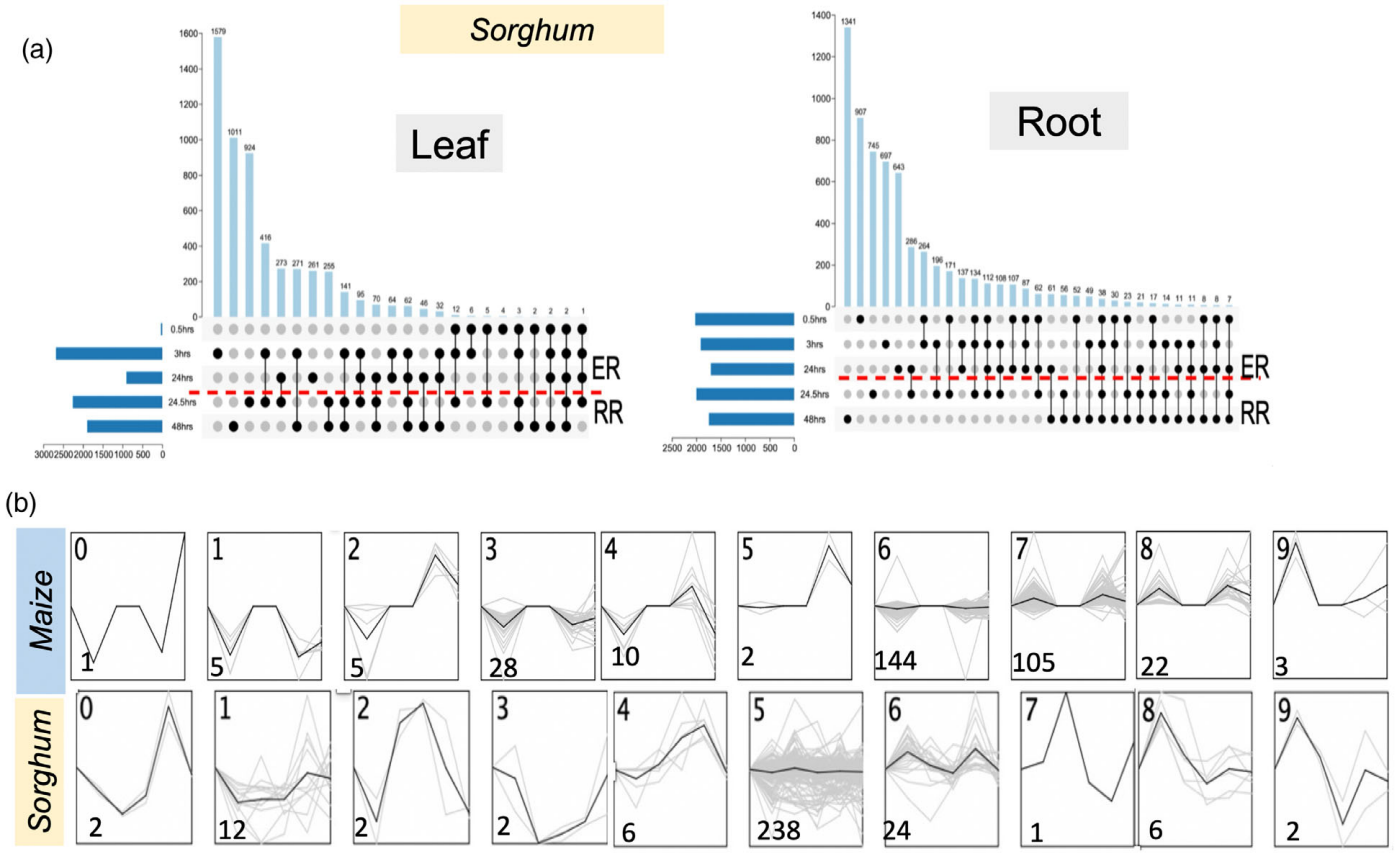
Spatiotemporal transcriptomic dynamics in maize and sorghum under varying nitrogen conditions. (a) UpSet plots depict the number of differentially expressed genes patterns in 0.5, 3, and 24 h under limiting nitrogen and at 24.5 and 48 h time points after recovery patterns in leaves and roots in sorghum. (b) Genes within the nitrogen uptake efficiency gene regulatory network were categorized into 10 clusters based on expression dynamics in maize and sorghum roots. Gray lines represent individual gene expression trajectories, while black lines serve as the representative expression profile for each cluster.

We extended our findings to another monocot species, *Sorghum bicolor*. In the absence of eY1H assays for sorghum, we developed its GRN using ortholog projections from maize, employing synteny analysis to identify 24 299 orthologous gene pairs. This method generated a projected network of 1596 PDIs, consisting of 93 promoters and 226 TFs for the sorghum NUE GRN (spNUEGRN) (Table S12; Figure 6, Figure S8). We further investigated sorghum gene expression in leaf and root tissues, focusing on responses at early N deprivation (0.5, 3, and 24 h) and recovery (24.5 and 48 h) conditions similar to our maize data. In the sorghum data, 35% of genes in leaves showed differential expression at 3 h, and 28% in lateral roots at 24.5 h, whereas in primary roots, 21% of DEGs at 0.5 h of N deprivation and during recovery at 24.5 h, mirroring similar trends observed in maize (Tables S13 and S14; Figure 7a,b).

The expression data were overlaid onto the network (Table S15) to assess if the expression patterns indicate putative conservation between the maize GRNs and the projected sorghum network. Gene expression data of maize and sorghum were then clustered into 10 distinct groups according to temporal profiles of the nodes within both the maize and sorghum networks (Figure 7b). Within the leaf data, it was revealed that N availability influenced expression trends in Clusters 0, 1, 2, and 5; specifically, a marked decrease in expression was observed between 0.5 and 3 h of N deprivation in both maize and sorghum (Table S16a,b). In maize, clusters 0, 1, 2, 3, and 5 demonstrated an initial decline in gene expression at the 0.5 h time point under N‐limiting conditions, followed by a further decrease at the 24‐h time point for root datasets (Figure 7c; Figure S9; Table S16c). In contrast, clusters 6, 8, and 9, in roots for maize showed opposing expression trends under 0.5 and 24 h suggesting initial responses to N deprivation are varied, but general trends emerge over time (Figure 7b). Notably, Clusters 0, 1, 2, and 5 in sorghum for roots showed patterns similar to those in maize, with an initial increase in gene expression under N‐limiting conditions (Table S16d). Despite these variations, no clusters favored specific N processes or TFs.

Using these data, we integrated Gini correlation analyses for maize and sorghum to gain insights into gene regulation patterns and TF interactions within the mNUEGRN and spNUEGRN networks. Given the extensive expression of N assimilation genes in maize, we began by examining these patterns in sorghum. In sorghum leaves, most nitrate reductase genes showed fewer correlated interactions, except for NR2, which demonstrated a negative correlation with NAC25. In contrast to maize, where bZIP34 interacts with GS1‐3 with a positive correlation, sorghum showed a negative correlation for this interaction. Additionally, glutamine synthesis‐related genes exhibited more differential expression in sorghum than in maize. In primary roots, we observed a consistent downregulation of both nitrate and nitrite reductase at the 3‐h time point under N deprivation, like patterns in maize but with timing differences. While both sorghum and maize showed mixed positive and negative correlations for nitrate reductase genes NR2 and NR3, some correlations diverged between species. For instance, in sorghum SbbZIP81, an ortholog of maize ZmbZIP18, was downregulated at the 0.5‐ and 3‐h time points during N deprivation and again at the 24.5‐h recovery phase, showing a negative correlation (Table S17), whereas maize ZmbZIP18 was not responsive to the N treatments but still functioned as a network hub. Additionally, SbOrphan207 in sorghum exhibited a species‐specific N response, with significant downregulation in primary roots at 0.5 h, contrasting with its upregulation in maize. In maize, Orphan207 interaction held significance as a regulator, while in sorghum, this was not the case.

**Figure 8:**
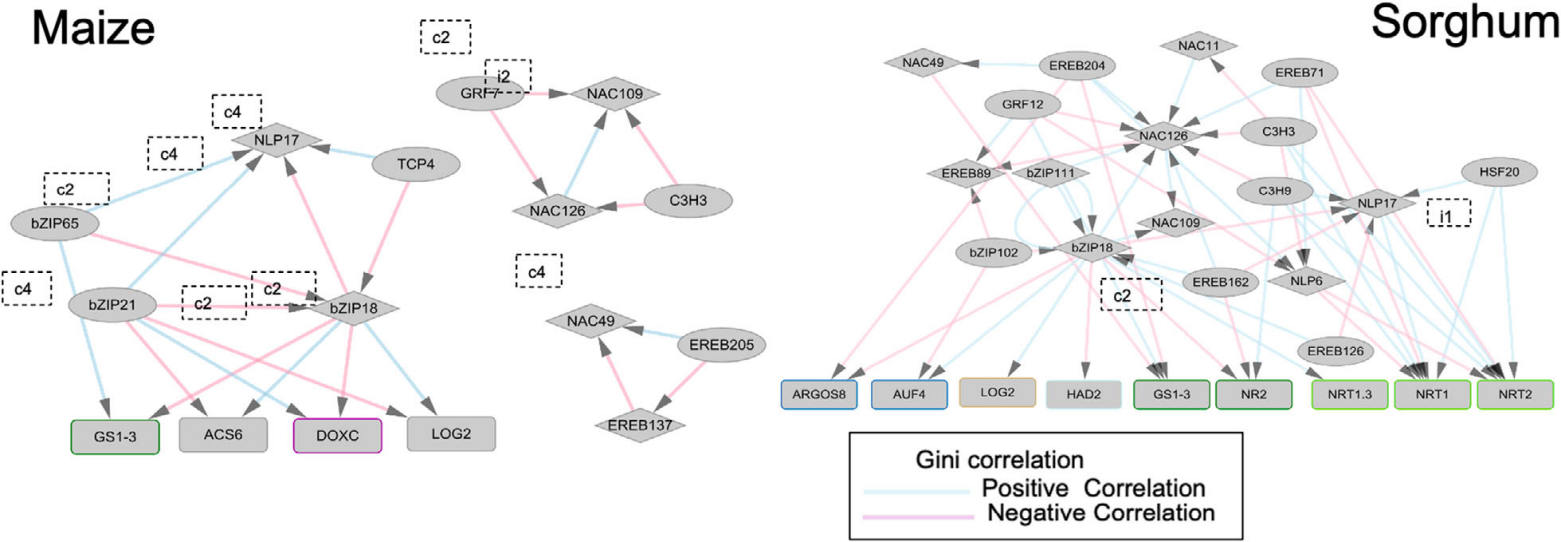
Feed‐forward loops in the transcriptional response to nitrogen stress in both maize and sorghum roots. Positive Gini correlations are represented in blue, and negative interactions are shown in pink.

However, within the sorghum network, we found that N transporters were more significant than in maize with interactions from NLP17 (SbNLP2), which are then modulated by HSF20 with a positive correlation. Similar to the maize network, we analyzed FFLs for the sorghum network, and we found a total of 638 FFLs (Table S18). Among those 638 FFLs, we found 9 significant FFLs for the leaf data and 26 for the root data based on Gini correlation (Figure S10). The interaction of SbNLP2 (ZmNLP17) was found to exhibit incoherent type 1 (i1) FFLs with HSF20 and NRT2.2 in the primary root (Figure 8). Further analysis of the FFL motifs within our sorghum datasets revealed unique evidence for regulatory rewiring of the SbbZIP81 as compared to its ortholog, ZmBZIP18, in maize. Traditionally, FFLs involved a TF regulating both a secondary TF and a downstream target gene. However, in sorghum, SbbZIP81 exhibited significant interactions that were more of a TF‐TF centric regulatory motif which deviates from what is seen in traditional FFLs. This deviation from traditional FFLs points to a reorganization of the transcriptional network within sorghum during N deprivation and N recovery. While maize ZmbZIP18 also appears to participate in similar FFL motif structures, the regulatory shifts in sorghum are more pronounced. These differences suggest a possible species‐specific adaptation in the regulatory architecture of bZIP18 and related transcriptional regulators though additional evidence including functional validation of key TF‐Target interactions and broader sampling across grass species would be needed to establish evolutionary trajectories with confidence. Together, the data suggest that although some core interactions are conserved, the regulatory roles diverge between maize and sorghum possibly reflecting lineage‐specific responses to environmental or developmental cues.

## DISCUSSION

Our study presents a comprehensive comparative analysis of the GRNs governing N metabolism in Arabidopsis, maize, and sorghum. These results not only deepen our understanding of the conserved and species‐specific regulatory elements in plant N metabolism but also offer valuable insights for future research. One of the most striking findings is the significant evolutionary conservation of specific key components and interactions within the GRNs across these species as measured by eY1H interactions and transcriptome profiling. Notably, the conservation of N assimilation pathway regulation by specific TFs such as bZIP family members, emphasizes the likely fundamental importance of these elements. This suggests that the primary architecture for N metabolism may have been established early in plant evolution and conserved across lineages, as previously proposed (Valverde et al., 2018). However, experimental validation is essential to confirm the functional significance of the identified regulatory interactions found in this research. Utilizing mutagenized lines, techniques such as chromatin immunoprecipitation (ChIP) or other heterologous assays can provide *in planta* validation for the elucidated networks.

### Distinct temporal responses to N deprivation and recovery

The close relationship between plant development and N availability, a key nutrient for plant growth, is well documented (Yousuf et al., 2022). Numerous studies have investigated plant responses to N availability, mapping essential genes and pathways involved in this complex process (Bi et al., 2007; Vidal et al., 2020). However, our study emphasizes the temporal dynamics of these responses, particularly during sudden changes in N availability in pathways such as N assimilation and transport, which appear more intricate than previously understood for maize and sorghum under early response to N deprivation. Maize and sorghum, both staple crops of substantial agronomic importance, exhibited rapid and extensive transcriptional reprogramming within just 0.5 h of N deprivation, emphasizing their evolutionary adaptation to fluctuating environments. Notably, while nitrate assimilation genes in both species showed broadly similar trends, we observed species and tissue‐specific differences in timing. For instance, we observed that leaf responses were more consistent, whereas root responses fluctuated and, in some cases, transitioned from downregulation to upregulation at later time points. These species‐specific differences also extend to N transporters and carbon metabolism. In maize, transporters such as NRT1.1, NRT1.3, AMT1.3, and AMT2.3 in leaves were repressed at early time points of N deprivation but rebounded strongly during recovery, indicating active reestablishment of nitrate flux. Carbon‐related enzymes such as PEPC and PGM also reset rapidly in maize leaves following N recovery. However, maize roots exhibited more upregulation during N recovery, suggesting tissue‐specific prioritization of recovery. In contrast, sorghum displayed a slower and more variable recovery in leaves for N transporters. While root nitrate transporters were upregulated early, both NRT1 and NRT2 were repressed at later stages (48 h), indicating a prolonged or delayed adjustment in sorghum. Sorghum carbon metabolism also diverged from maize; G6PD1 expression in maize was affected only at 3 h of N deprivation, when it was transiently downregulated in roots, whereas in sorghum, it was consistently downregulated at 0.5–24 h in roots during N deprivation. Interestingly, in sorghum, G6PD1 expression from 3 to 48h in leaves was downregulated. However, PEPC and PGM showed little transcriptional response to the N deprivation and recovery. These findings suggest that maize employs a rapid reset strategy to restore nitrogen and carbon homeostasis, while sorghum adopts a more gradual, adjustment to changes in N status. These patterns highlight finely tuned, temporally staged regulatory systems for nutrient sensing and response. This aligns with earlier research on the short‐term dynamics of plant transcriptomes in response to environmental stresses, such as drought or nutrient stress (Menz et al., 2016; Singh et al., 2022).

### Key TFs and network stability

One striking finding from our study is the divergence in expression profiles of specific TF families, namely members of the bZIP and EREB families between the closely related crops, maize and sorghum. Despite their close phylogenetic relationship, the distinct expression trajectories suggest adaptations specific to each species, possibly driven by differences in native habitats or domestication histories. The bZIP TF family, in particular, has been implicated in various stress responses, including nutrient deprivation (Erpen et al., 2018; Golldack et al., 2014). The distinct patterns we observed between maize and sorghum may reflect their unique evolutionary strategies for managing N fluctuations. Where maize generally requires more N due to its higher yield potential and greater N uptake, sorghum, being more drought tolerant, has lower N requirements, especially under water‐limited conditions (Muchow, 1998). As our treatments did not include water‐limited conditions for either maize or sorghum, we observed distinct shifts in TFs expression patterns, consistent with the changing N requirements of maize and sorghum.

TFs central in the network reveal their potential role as master regulators, controlling multiple downstream target genes associated with N responsiveness. By binding to a broad range of promoter regions, these TFs act as hubs, disseminating signals to orchestrate a coordinated response to N fluctuations in multiple biological processes (Du et al., 2020). This is particularly relevant in complex pathways, such as N transport, adding another layer of complexity to our understanding of plant N metabolism and its adaptability to changing environments. Within this framework, ZmNLP17 emerged as a particularly influential regulator in our GRN. Its extensive connectivity, including interactions with nitrate uptake and assimilation genes as well as other TFs, positions it as a central hub in maize N metabolism. This observation aligns with independent evidence from Cheng et al. (2021), which identified NLP17 as one of the top‐ranked TFs predictive of N use efficiency in field‐grown maize. Moreover, functional studies of its Arabidopsis homolog, NLP5, demonstrated that loss‐of‐function mutants exhibit enhanced N use efficiency under low‐N conditions, supporting the idea that NLP17 integrates N sensing with transcriptional responses. Together, these results suggest that NLP17 plays a pivotal role in coordinating N‐responsive regulatory networks in maize. Additionally, our network analysis highlights a regulatory module centered on NLP17 that connects to other key TFs, notably bZIP30 and bZIP18. Both bZIP TFs show extensive connectivity in the maize and sorghum GRNs, with bZIP18 directly targeting NAR2 in the sorghum leaf network and bZIP30 forming multiple interactions with NLP17/NLP6. These links suggest that bZIP18 and bZIP30 may act as integrators of N signaling, bridging upstream nitrate sensing with downstream transcriptional responses. Their functional relevance is further underscored by their evolutionary conservation: maize bZIP18 and bZIP30 are orthologs of Arabidopsis GBF proteins, which are known regulators of multiple stress‐responsive pathways, including N deprivation. Moreover, overexpression of maize bZIP30 in rice has been shown to enhance plant performance under stress (Perveen et al., 2020), providing additional support for their roles as positive regulators of N‐associated responses. Together, these findings position NLP17, bZIP18, and bZIP30 as a potential core regulatory module coordinating N uptake, assimilation, and adaptation across species.

Our findings reveal a complex regulatory network characterized by what can best be described as ‘consistent rewiring’ within these systems. A particularly challenging aspect of our study was identifying the specific TFs responsible for regulating key transporters involved in N uptake and metabolism. This difficulty may stem from the multifaceted nature of these networks, where multiple TFs often interact to co‐regulate target genes (Swift & Coruzzi, 2017). Notably, we observed instances where TFs not only interacted with the promoter regions of transporter genes but also with the promoter regions of other TFs, adding an additional layer of complexity to the already intricate regulatory landscape. To address this complexity, we employed the NECorr package and the Gini correlation coefficient to study co‐expression relationships. These tools provide a robust framework for disentangling the multifaceted regulatory relationships, providing deeper insights into gene regulation (Ko & Brandizzi, 2020).

In GRNs, FFLs are important for coordinating responses to changes in nutrient availability and environmental conditions. By integrating inputs from different TFs, FFLs help fine‐tune metabolic processes and stress adaptation. FFLs are broadly classified into eight categories (coherent types I–IV and incoherent types I–IV), each defined by the direction and nature of the regulation. In our analysis, Gini coefficients suggesting activation and repression were used to identify the types of FFLs present. Within maize, interactions among bZIP TFs illustrate the complexity of these regulatory circuits. For example, bZIP18, together with bZIP30 and related family members, was associated with incoherent type IV and coherent type II FFLs. These configurations may contribute to pulse‐like expression dynamics in pathways such as nitrate assimilation and N reductase. In sorghum, however, we observed different FFL patterns, with coherent type I and type III more prominent. This contrast suggests possible species‐specific differences in regulatory architecture, though the extent of functional conservation remains to be determined. Direct characterization of bZIP18 mutants in both maize and sorghum will be necessary to clarify whether these observed network patterns reflect conserved functional roles.

By engaging in coherent and incoherent FFLs, these TFs orchestrate gene expression patterns that are crucial for maintaining cellular homeostasis and promoting adaptive responses. This sophisticated regulatory architecture underscores the importance of understanding FFLs to decipher the broader principles of gene regulation in plants. Notably, NAC108 emerges as a central node within a leaf FFL targeting *TIR1* and auxin signaling. Given NAC108's established role in senescence and photosynthetic apparatus degradation, its placement in this circuit points to a context‐dependent function in which NAC108 may couple nitrogen status with hormone‐mediated developmental transitions. This suggests that NAC108 integrates metabolic and degradation pathways, reinforcing the idea that FFLs provide flexible platforms for cross‐tissue and cross‐process coordination. Overall, the study of FFLs in GRNs not only enhances our comprehension of basic biological processes but also offers potential applications in agriculture and biotechnology. By manipulating these regulatory circuits, it may be possible to develop crops with improved resilience to environmental stresses and enhanced nutrient use efficiency.

## CONCLUSION

This study serves as a comprehensive exploration into the GRNs governing N metabolism across Arabidopsis, maize, and sorghum. It brings forth key insights that deepen our understanding of the evolutionary conservation and divergence in the regulation of NUE, a critical factor in sustainable agriculture. We leveraged an array of methodologies including Y1H assays, DAP‐seq techniques, and ortholog‐based strategies, thereby uncovering high‐confidence PDIs and revealing the role of TFs in N metabolism. The study outlines both the conserved and unique elements of these GRNs, drawing attention to the evolutionary plasticity within each species due to genome duplication events and environmental pressures as is the case for the maize genome. While the core architecture for N metabolism is conserved across species, our study reveals species‐specific variations in the fine‐tuning and rewiring of GRNs. These variations contribute to the adaptability of plants to specific environmental challenges. Our results emphasize the importance of temporal dynamics in the N response of maize and sorghum. Both crops exhibit intricate adaptations that enable them to swiftly respond to fluctuations in N availability. These findings highlight the distinct, yet finely tuned strategies employed by maize and sorghum to reset their metabolic balance during periods of N deprivation and recovery, emphasizing the robust resilience of these plants. In conclusion, our study provides a comprehensive framework for understanding the complex regulatory landscape of N metabolism in plants. These insights have implications for both basic science and applied research, offering potential avenues for enhancing crop yield and environmental sustainability.

## MATERIALS AND METHODS

### Plant materials and growth conditions

Maize B73 and Sorghum BTx623 seeds were first treated with fungicides, bleach, ethanol, and deionized water (dH_2_O). The treated seeds were germinated in the moistened rolls in a growth chamber under a 16‐h light/8‐h dark cycle, with temperatures set at 30°C/23°C. The growth chamber provided 600 μmol m^−2^ sec^−1^ illumination and maintained 50% humidity. After germination, seedlings with consistent root emergence were selected: maize after 3 days and sorghum after 7 days. These seedlings were then transferred to Magnavaca's nutrient solution, optimized for normal nitrogen (NN) treatment (Magnavaca et al., 1987).

After 12 days of growth in the NN solution, both maize and sorghum plants were transferred to a modified Magnavaca's nutrient solution with limiting nitrogen (LN) treatment. In this solution, Ca(NO_3_)^2^ and MnSO_4_ were replaced with CaCl_2_ and MnCl_2_, and all nitrate salts were removed from the medium. Root and leaf samples from both species were harvested under the limiting nitrate conditions at intervals of 0, 0.5, 3, and 24 h. Additionally, after 1 day of nitrate deprivation, nitrates were reintroduced into the nutrient solution to observe nitrate recovery, with further root and leaf sampling at 0.5 and 24 h post‐initiation of recovery.

For leaf sampling, we collected the second and third fully developed leaves from both maize and sorghum during their respective fifth leaf stages. For root samples, sorghum primary and lateral roots were collected separately, while for maize, seminal and crown roots were collected and mixed. To ensure a representative sample for each time point, 10–15 plants were pooled together. Additionally, three biological replicates were conducted for both leaves and roots for each species, providing robust data for analysis.

### Establishing a GRN for maize using Y1H assay

To construct a comprehensive GRN for maize, our study began with the identification of key target genes and TFs associated with N metabolism. We used our previous work on the Arabidopsis on an enhanced *Y1H* assay GRN outlined by Gaudinier et al. (2018) as a foundational model for the comparative genomics on maize and sorghum. The Arabidopsis Y1H GRN included 1660 PDIs comprising 345 TFs and 98 promoters. Using an ortholog‐based approach through Gramene compara from the maize PanGenome release 2.0 (Tello‐Ruiz et al., 2022), we integrated the maize orthologs of Arabidopsis eY1H GRN. Additionally, we incorporated additional TFs on maize N regulation from the published studies which allowed us to increase the percentage of TF promoters (baits) related to N processes. In assembling the Y1H assay network of maize, we cloned and transformed selected TFs. Specifically, 104 maize promoters from B73 maize genomic DNA were isolated, cloned, and amplified to 2 kb or to the nearest upstream gene. These were recombined into pDONRP4‐P1R entry vectors using Phusion Taq polymerase (NEB), while others were synthesized and cloned into an *attL4–attR1* vector backbone. The recombined promoters were fully sequenced and then transferred into reporter vectors pMW2 and pMW3 using LR Clonase II (Invitrogen, Carlsbad, CA, USA). These constructs were sequence‐confirmed and then introduced into the yeast strain YM4271. Additionally, constructs that were resistant to transformation in YM4271 were instead transformed into the Y1H‐S2 strain. Moreover, 493 TFs, sourced from the maize TFome, were amplified and recombined into D‐TOPO (Invitrogen), sequenced for accuracy, and then recombined into pDEST‐AD‐2μ using LR Clonase II (Invitrogen). These constructs were transformed into the yeast strain Yα1867 following the protocol outlined by Gaudinier et al. (2018). Yeast colonies were screened for autoactivation and construct presence, and the promoter strains were mated against the TF strains as previously described.

### Comparison of maize Y1H network with Arabidopsis DAP‐seq data

To assess the conservation of PDIs between maize and *A. thaliana*, we compared the maize Y1H network with Arabidopsis DAP‐seq data and the existing Arabidopsis Y1HGRN used to construct the mNUEGRN (O'Malley et al., 2016). Using an orthology‐based approach, we identified maize orthologs of Arabidopsis TFs and target genes via Gramene Compara (Tello‐Ruiz et al., 2022) and examined whether the regulatory interactions observed in maize were conserved in Arabidopsis. We integrated DAP‐seq TF binding profiles from Arabidopsis, mapping TF binding sites onto maize promoter regions to assess overlap with maize Y1H‐derived interactions. This analysis identified a subset of PDIs that were conserved across both species, highlighting core regulatory elements in N metabolism. The conserved interactions were not used to filter the maize GRN but rather to determine which regulatory relationships were shared across species and which lacked corresponding DAP‐seq evidence from Arabidopsis datasets. The final network visualization was generated using Cytoscape v3.9.0 (Shannon et al., 2003), allowing for an in‐depth comparison of species‐specific versus evolutionarily conserved N regulatory interactions.

### Network connectivity analysis

To assess the connectivity and regulatory influence of TFs within the maize N GRN, we calculated the expected outdegree for each TF. The outdegree represents the number of target genes regulated by a given TF, providing insight into the distribution of regulatory interactions across the network.

From this network, the expected outdegree for analysis and significance of TF families within modules was calculated as follows:

$$\text{Expected Outdegree for}\;\mathrm{TF}=\begin{array}{l} \frac{\text{Number of}\;\mathrm{TF}\;\text{Members}}{\text{Total Number of}\;\mathrm{TFs}}\\ \times \;\text{Total Outgoing Edges.} \end{array}$$

The chi‐square statistic is then calculated using:

$$\chi^{2}=\sum \left(\frac{{\left(O_{i}-E_{i}\right)}^{2}}{E_{i}}\right)$$

where (*O*_*i*_) is the observed frequency and (*E*_*i*_) is the expected frequency, derived from the calculation above. This test assesses the deviation of observed interactions from those expected under a hypothesis of random distribution across the network. The *P*‐value, corresponding to the chi‐square statistic and reflecting the probability of observing such a deviation by chance, is calculated based on the chi‐square distribution with degrees of freedom set to 1 (for the comparison of one category).

### RNA‐Seq library construction and data analysis

We used specific RNA extraction methods which were tailored for maize and sorghum. In maize, total RNA was isolated from hydroponically grown samples of root and leaf collected at various time points: 0.5, 3, 24, 24.5, and 48 h. These time points were chosen to capture early, intermediate, and long‐term transcriptional responses to N depletion and resupply. This process involved the use of TRIzol reagent, followed by RQ1 DNase treatment, phenol/chloroform/iso‐amyl alcohol extraction, and ethanol precipitation (Rio et al., 2010). For sorghum, the total RNA extraction from samples at corresponding time points (mentioned above) was performed using the Zymo Research RNA/DNA mini purification kit, with minor adjustments to the standard protocol (Zymo Research, Irvine, CA, USA). In both species, we isolated poly(A)+RNA using Invitrogen oligo(dT) magnetic beads (610‐02). Subsequently, RNA‐seq libraries were prepared using the Epicentre ScriptSeq kit (SS10906) for maize and NEBNEXT kits from New England Biolabs for sorghum. These libraries were then sequenced on an Illumina HiSeq 2500 platform, generating raw sequence data in FASTQ format.

Quality control checks on these raw sequence files were carried out using FastQC (Andrews et al., 2010). The maize reads did not require trimming; the sorghum reads were trimmed using Trimmomatic v.0.3.9 (Bolger et al., 2014). After processing, the reads were aligned to their respective reference genomes, maize B73v5 and sorghum BTx623 v3.1, using the STAR aligner (Dobin et al., 2013). Transcript assembly was then performed using StringTie v2.1.4 with default parameters, and data were normalized in TPM (Pertea et al., 2015).

DEGs were identified using the DESeq2 R package, applying an adjusted *P*‐value threshold of less than 0.05 and a log2 Fold Change (log2FC) greater than 1. Functional enrichment analysis of these DEGs was conducted with the BINGO plug‐in in Cytoscape (Maere et al., 2005), focusing on Gene Ontology (GO) (Gene Ontology Consortium, 2021) and KEGG pathway enrichment (Kanehisa & Goto, 2000). Visualization of the results was done in RStudio, using the ggplot2 and enrichKEGG packages, with an emphasis on the top 20 significant molecular function/biological process GO terms and KEGG pathways.

### Developing a projected GRN for sorghum using orthology‐based comparative genomics

Similar to the identification of nodes in the maize GRN, we used an orthology‐based approach using the Gramene Compara pipeline to construct a corresponding NUE GRN for sorghum. Initially, the protein‐coding sequences of both maize and sorghum gene models, as available on Gramene, were subjected to a comparative analysis using the Ensembl Compara pipeline v107, to identify orthologs between the two species (Cunningham et al., 2022; Vilella et al., 2009). This method reports both one‐to‐one and one‐to‐many orthology relationships between maize and sorghum. Subsequent to the ortholog identification, a gene‐to‐gene synteny analysis was performed. For this purpose, we used DAGchainer, a tool for identifying conserved genomic regions and syntenic blocks across different species (Haas et al., 2004). Finally, syntenic orthologs were used to project the maize Y1H GRN to spNUEGRN.

### Gini correlation

Gini correlation coefficients were calculated between all PDI gene pairs using normalized gene expression data from the root and leaf samples for all the time points described previously for both maize and sorghum (Ma & Wang, 2012). Edges with a Gini correlation coefficient >0.5 or <−0.5 were considered activating or repressing, respectively.

### NECorr

The R package, NECorr, was employed to discern crucial regulator–gene interactions within our network. This method underscores the significance of hub genes in driving systemic cascades, as detailed by Liseron‐Monfils et al. (2018). The model is bifurcated into two main components: NECorr‐Hub and NECorr‐reg. NECorr‐Hub aggregates molecular network structure with gene expression from our RNA‐seq libraries, produced from the time‐series data, to prioritize genes based on their network significance. This is achieved by evaluating various parameters, including co‐expression based on the Gini coefficient across edges and tissue‐specific gene expression using the Gini coefficient. Such a ranking facilitates the identification of hub genes, instrumental in eliciting the right system responses. Subsequent to pinpointing these hub genes, the NECorr‐reg utilizes machine learning, specifically the random forest algorithm, to identify potential regulators linked to these hubs. Together, NECorr aims to highlight the regulatory dynamics inherent to molecular networks.

## AUTHOR CONTRIBUTIONS

DW, SMB, and LK conceived and designed the study. JB, LZ, MR, AG, A‐MB, BS, MJF, and SA conducted the experiments. JB, AO, and CL‐M constructed the networks for both maize and sorghum. JB, SK, and VK performed the data analysis. JB, SK, VK, and AO wrote the manuscript. DW, SMB, LK, SK, VK, AG, AO, and JB edited and refined the manuscript. All authors have reviewed and approved the final version.

## CONFLICT OF INTEREST

The authors have not declared a conflict of interest.

## Supporting information


**Figure S1.** Schematic diagram of the research workflow.
**Figure S2.** Overview of maize gene regulatory network statistics.
**Figure S3.** Network statistics of connectivity of TF families within the maize GRN.
**Figure S4.** Carbon metabolism in maize leaf and roots.
**Figure S5.** Nitrogen transporter module in maize leaf and roots with expression datasets.
**Figure S6.** Comparative analysis of conserved functional categories between Arabidopsis and maize networks.
**Figure S7.** Comparative analysis of G‐box binding factors in Arabidopsis and maize.
**Figure S8.** Visualization of the projected sorghum nitrogen uptake use efficiency gene regulatory network.
**Figure S9.** Temporal transcriptomic dynamics in maize and sorghum under varying nitrogen conditions.
**Figure S10.** Significant FFLs in sorghum leaf and roots.

**Table S1.** Promoter clones for the maize Y1H assays and TF (Libraries) clones for the maize Y1H assays.

**Table S2.** Maize NUE gene regulatory network (mNUEGRN).

**Table S3.** Network statistics for TF families, promoters and subfunctional process in the mNUE GRN.

**Table S4.** Indegree and outdegree information for large TF families within the mNUEGRN.

**Table S5.** Feedforward loops within the mNUEGRN.

**Table S6.** Differentially expression genes across all time points in leaf in maize.

**Table S7.** Differentially expression genes across all time points in roots in maize.

**Table S8.** Functional enrichment using gene ontology among leaf and roots for maize expression data.

**Table S9.** Differential expressed genes of leaf and roots in maize with functional description for promoters/TFs within the GRN.

**Table S10.** Gini correlation analysis in leaf and roots of maize.

**Table S11.** Conserved interaction in mNUEGRN in comparison to the Arabidopsis GRN.

**Table S12.** Nodes and edges within the sorghum projected gene regulatory network (spNUEGRN).

**Table S13.** Differentially expressed genes across all time points in leaf of sorghum.

**Table S14.** Differentially expressed genes across all time points in roots of sorghum.

**Table S15.** Differential expressed genes of leaf and roots in sorghum with functional description for promoters/TFs within the GRN.

**Table S16.** Clustering of the expression data (TPM) from leaf and root for maize and sorghum GRN.

**Table S17.** Gini correlation analysis in leaf and roots for sorghum.

**Table S18.** Feed‐forward loops of spNUEGRN.

## Data Availability

The data that supports the findings of this study are available in the Supporting Information of this article. Raw sequencing datasets generated in this study have been deposited to NCBI SRA under the accession number PRJNA883413. Additional scripts used for transcriptomics analysis related to this paper can be found on GitHub: https://github.com/JanBraynen/RNA‐seq_analysis. Additionally, we added all TPM values to NCBI GEO under the accession numbers GSE312408 and GSE312411 for maize and sorghum, respectively.
