# Supplementary material for: Decoding nitrogen uptake efficiency in maize and sorghum: insights from comparative gene regulatory networks
Source: Plant J. 2025 Dec 18;124(6):e70631. doi: 10.1111/tpj.70631 (PMC12714369; doi:10.1111/tpj.70631)
Supplement: Supplementary file 1 — Figure S1. Schematic diagram of the research workflow. Figure S2. Overview of maize gene regulatory network statistics. Figure S3. Network statistics of connectivity of TF families within the maize GRN. Figure S4. Carbon metabolism in maize leaf and roots. Figure S5. Nitrogen transporter module in maize leaf and roots with expression datasets. Figure S6. Comparative analysis of conserved functional categories between Arabidopsis and maize networks. Figure S7. Comparative analysis of G‐box binding factors in Arabidopsis and maize. Figure S8. Visualization of the projected sorghum nitrogen uptake use efficiency gene regulatory network. Figure S9. Temporal transcriptomic dynamics in maize and sorghum under varying nitrogen conditions. Figure S10. Significant FFLs in sorghum leaf and roots. [file TPJ-124-0-s007.docx]

**Decoding Nitrogen Use Efficiency in Maize and Sorghum: Insights from Comparative Gene Regulatory Networks**

Janeen Braynen^1^, Lifang Zhang^1^, Sunita Kumari^1^, Andrew Olson^1^, Vivek Kumar^1^, Michael Regulski^1^, Christophe Liseron-Monfils^2^, Allison Gaudinier^3,4^, Anne-Maarit Bågman^5,8^, Shane Abbitt^6^, Mary J. Frank^6^, Bo Shen^6^, Leon Kochian^7^, Siobhan M. Brady^5,8^, Doreen Ware^1,9^

^1^ Cold Spring Harbor Laboratory, 1 Bungtown Road, Cold Spring Harbor, NY 11724.

^2^ National Research Council Canada, 101 gymnasium place, Saskatoon, SK, S7N 0W9, Canada.

^3^ Department of Plant and Microbial Biology, University of California Berkeley, Berkeley, CA 94720.

^4^ Miller Institute for Basic Research in Science, University of California Berkeley, Berkeley, CA 94720.

^5^ Department of Plant Biology and Genome Center, University of California Davis, Davis, CA, 95616.

^6^ Corteva Agriscience, 7300 NW 62nd Avenue, Johnston, IA, 50131 USA

^7^ Department of Plant Sciences, University of Saskatchewan, Saskatoon, SK S7N4L8, Canada.

^8^ Howard Hughes Medical Institute, University of California, Davis, Davis CA 95616.

^9^ USDA-ARS-NAA, Ithaca, NY 1485.

Supplementary Figures


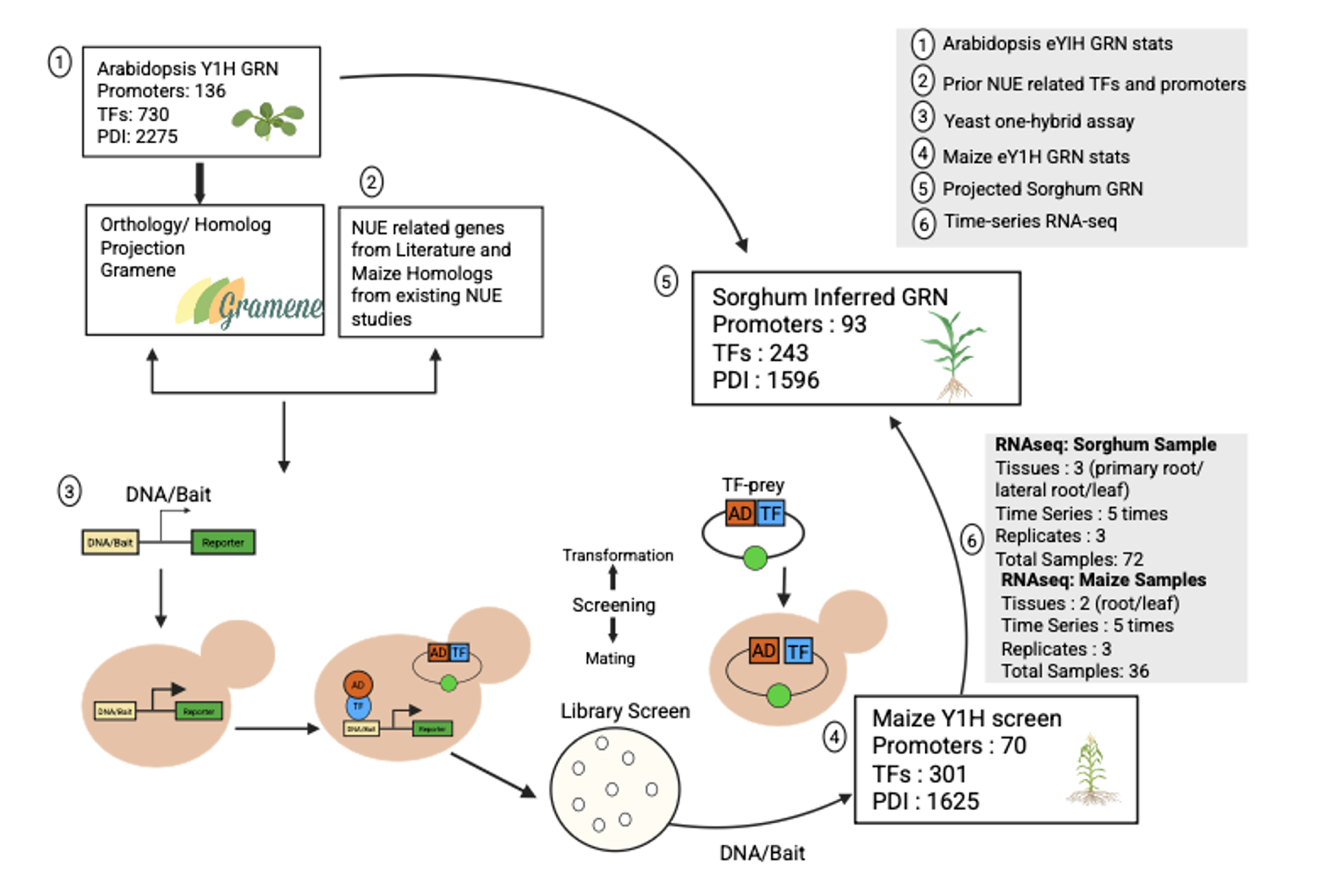


**Figure S1** **Schematic Diagram of the Research Workflow** This schematic diagram illustrates the key steps in constructing a gene regulatory network from ortholog prediction using Y1H assays in *Arabidopsis*, leveraging the Gramene infrastructure. It includes the integration of nitrogen use efficiency (NUE)-related genes, identified through a comprehensive literature review of genes commonly associated with nitrogen metabolism in maize and other crop species. Following ortholog prediction, Y1H assays were performed to construct the gene regulatory network, which was then compared and overlapped with gene expression data. Numbers in the diagram correspond to specific stages of the experimental workflow. The Y1H assay representation has been adapted from Sewell and Furman Bass (2018). In boxes 1, 4 and 5 in Figure S1, PDI stands for protein-DNA interaction.


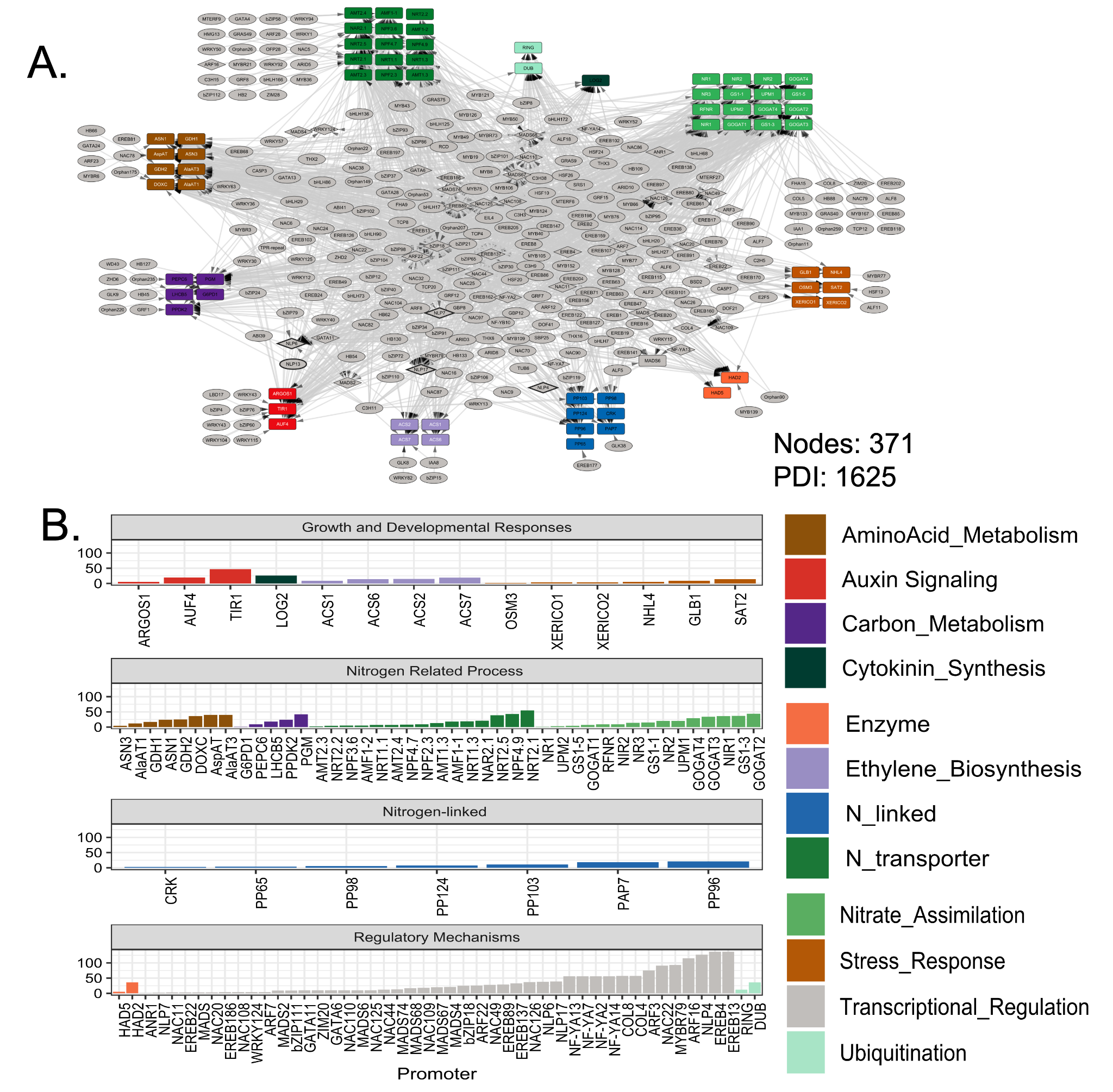


**Figure S2** **Overview of Maize Gene Regulatory Network Statistics.** (A) The Cytoscape visualization presents maize sub-networks by functional categories and transcription factors (TFs), with nodes depicted as ovals for TFs (prey), rectangles for promoters (baits), and diamonds for entities serving as both TFs and promoters. (B) The number of TFs binding to promoters across these four promoter groups: growth and development, nitrogen-related, nitrogen-linked, and regulatory mechanisms. Different shapes represent different nitrogen processes: rectangles for promoters, ovals for transcription factors (TFs), and diamonds for genes functioning both as promoters and TFs. Distinct colors highlight various nitrogen-associated processes: light green for nitrogen transport, dark green for nitrate assimilation, purple for amino acid biosynthesis, pink for stress response, red auxin signaling, brown ethylene biosynthesis, blue nitrogen linked, light blue enzymes, orange carbon metabolism, dark brown ubiquitination and yellow cytokinin synthesis. Genes with blue borders represent orthologs, while those with red borders signify homologs of Arabidopsis.


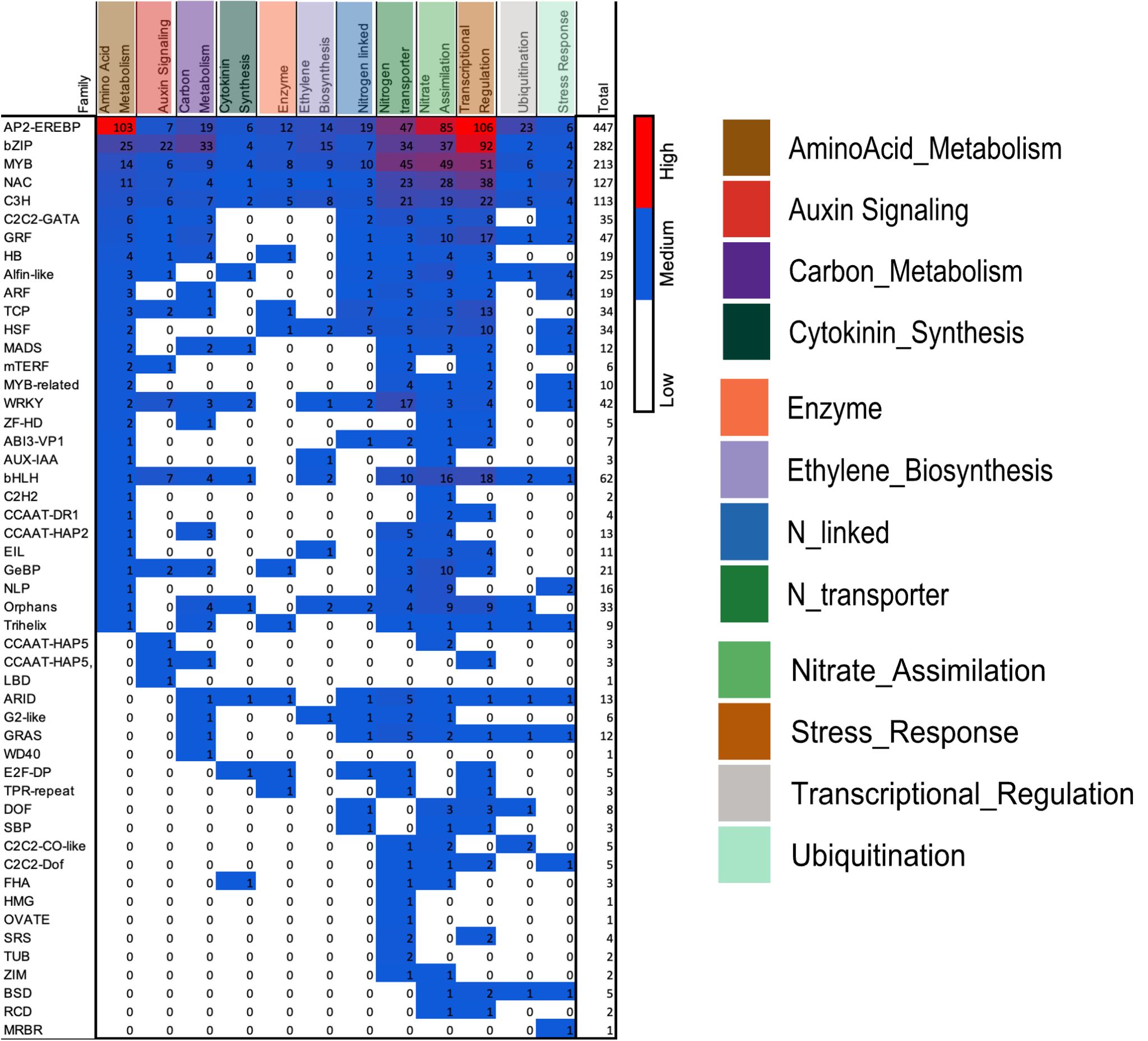


**Figure S3** Network statistics of connectivity of TF families within the Maize GRN. Color scale based on the number of connections within each submodule.


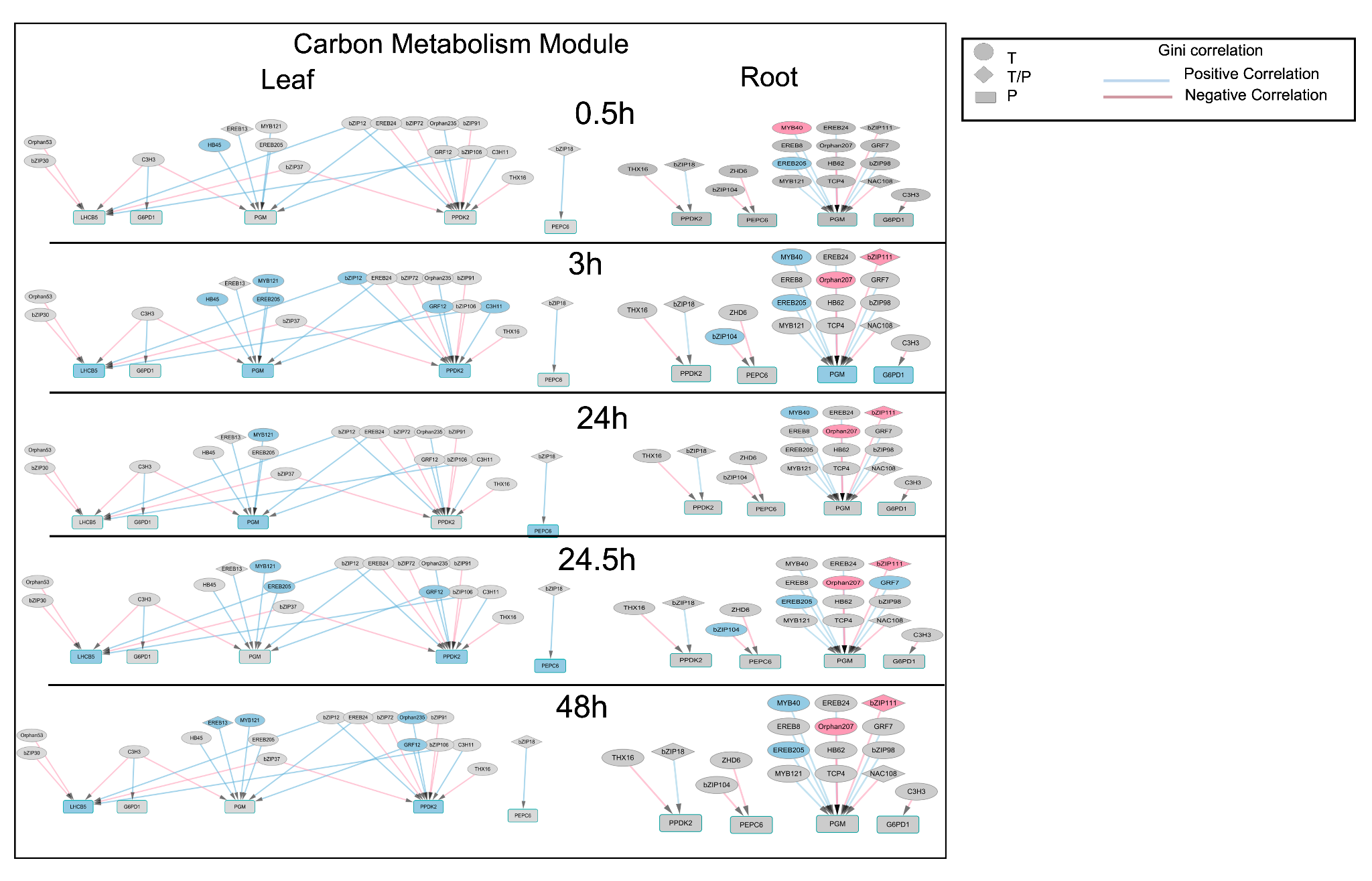


**Figure S4 Carbon Metabolism in Maize Leaf and Roots.** Time series transition data illustrating dynamic changes in carbon metabolism over specified time points, with mini correlations mapped onto the carbon metabolism pathway. Upregulated genes are indicated in pink, while downregulated genes are shown in blue. Positive Gini correlations are represented in blue, and negative interactions are shown in pink. Gini correlation matrix showing correlation >0.5 or <-0.5 of the leaf and roots.

**
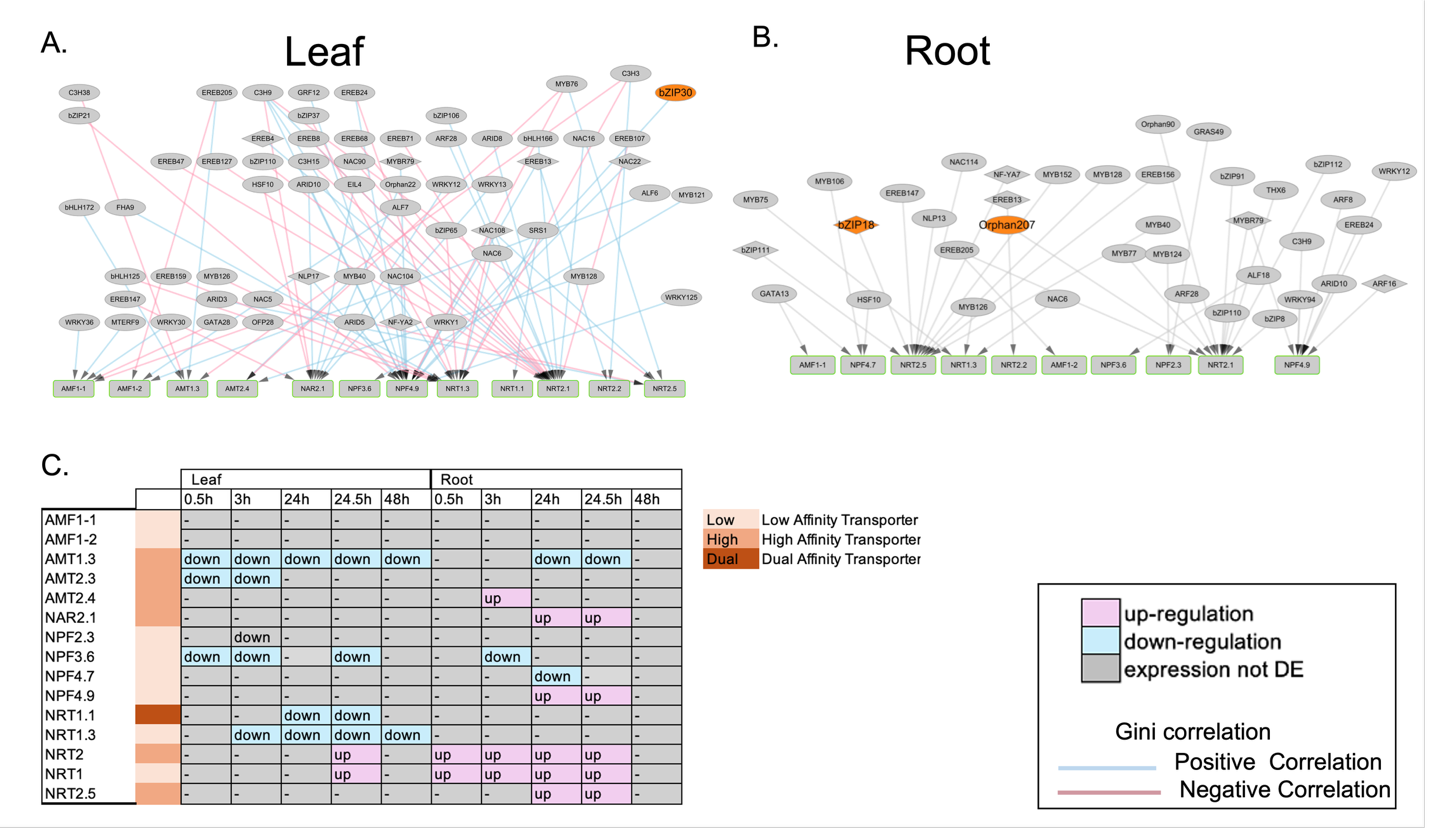
**

**Figure S5 Nitrogen Transporter Module in Maize Leaf and Roots with Expression Datasets.** (A-B) Nitrogen (N) transporter module displaying mini correlation for leaf and root tissues, respectively, indicating network interactions within nitrogen transport pathways. (C) Expression patterns of nitrogen transporters in maize leaf and root samples. Upregulated genes are indicated in pink, while downregulated genes are shown in blue. Positive Gini correlations are represented in blue, illustrating supportive interactions, and negative interactions are shown in pink, highlighting inverse relationships within the module.

**
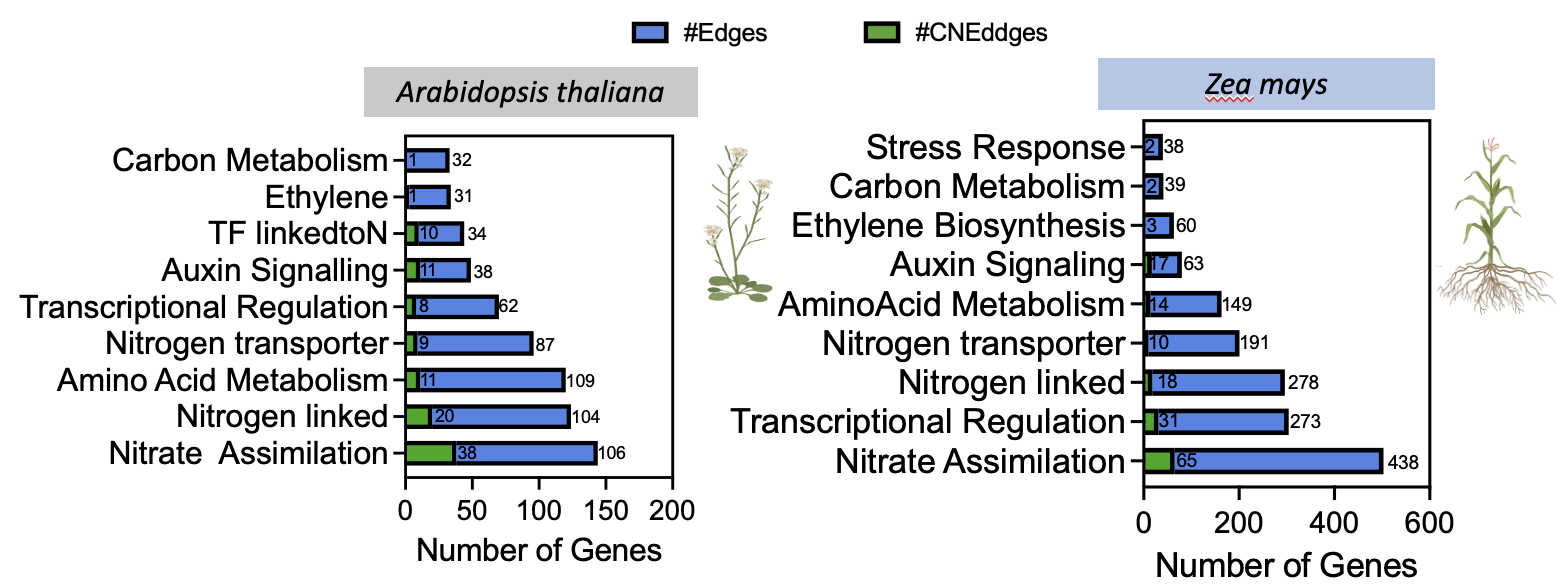
**

**Figure S6 Comparative analysis of conserved functional categories between Arabidopsis and maize networks.** (a-b) These sections highlight the conserved interactions focusing on nitrogen-related processes. The green section specifically emphasizes the conserved protein-DNA interactions (PDIs) within this framework.


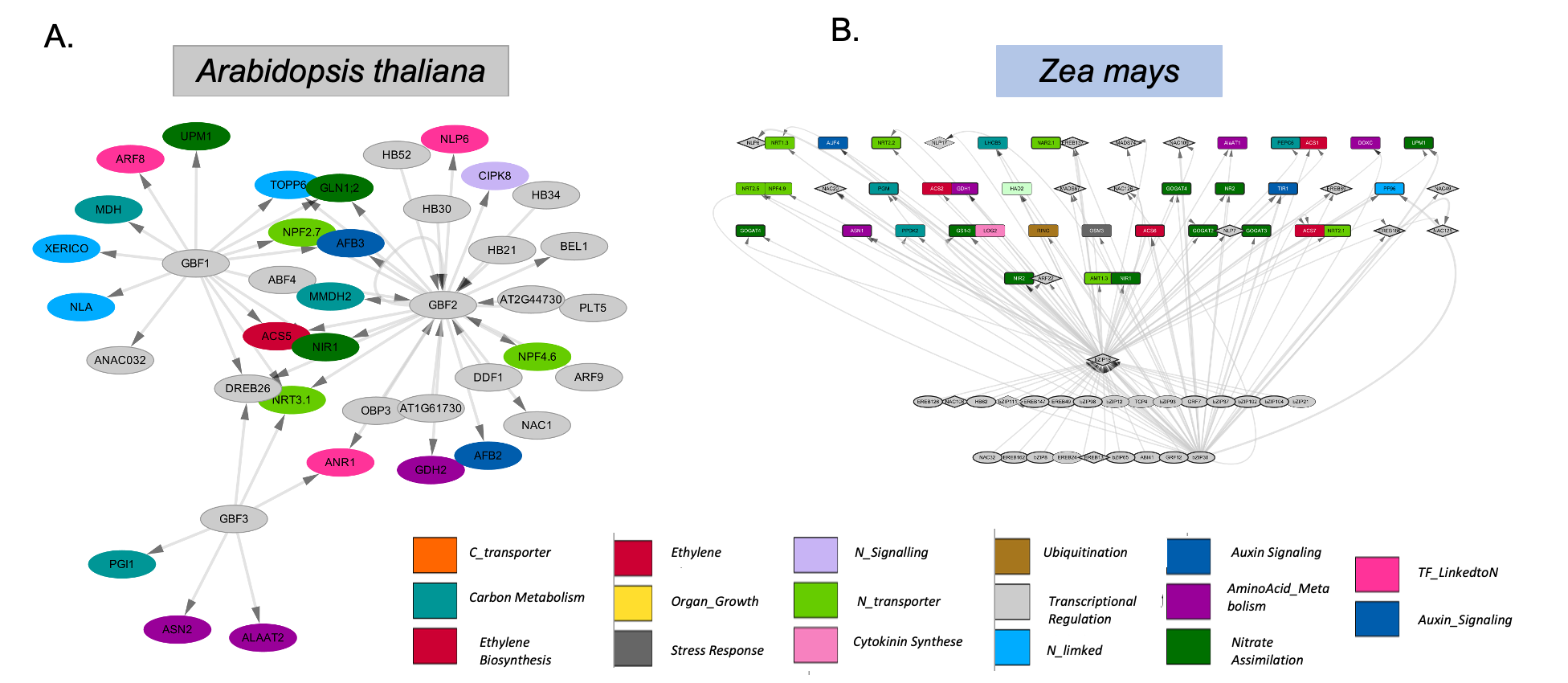


**Figure S7 Comparative analysis of G-box binding factors in Arabidopsis and Maize.** (a) In Arabidopsis, GBF1, GBF2, and GBF3, critical for stress-responsive networks, exhibit conserved interactions primarily in multiple nitrogen pathways. (b) The Maize orthologs of Arabidopsis GBF TFs, bZIP18 and bZIP30, demonstrate hierarchical interactions, reflecting the complex regulatory networks in Maize.


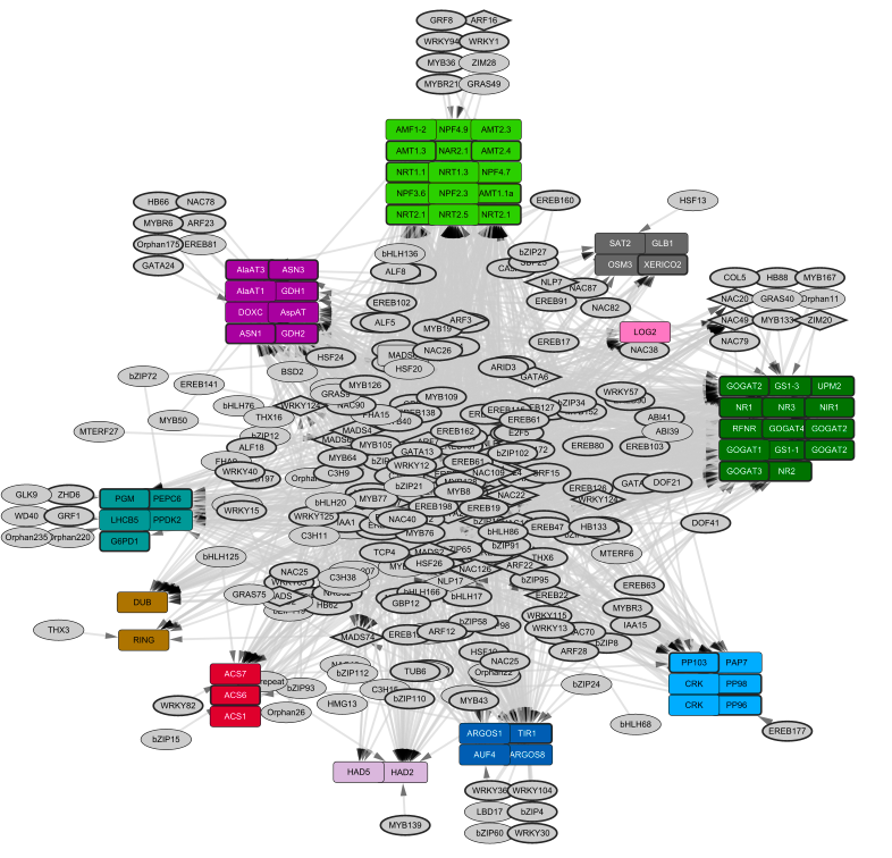


**Figure S8 Visualization of the Projected Sorghum Nitrogen UptakeUse Efficiency Gene Regulatory Network.** Various shapes and colors denote different components and processes: rectangles indicate promoters, ovals represent TFs, and diamonds symbolize genes that function as both promoters and TFs. The colors designate specific nitrogen-associated processes: light green for nitrogen transport, dark green for nitrate assimilation, purple for amino acid biosynthesis, gray for stress response, blue for auxin signaling, lavender for enzyme activity, red for ethylene, brown for ubiquitination, light blue for N-linked glycosylation, and pink for cytokinin synthesis.


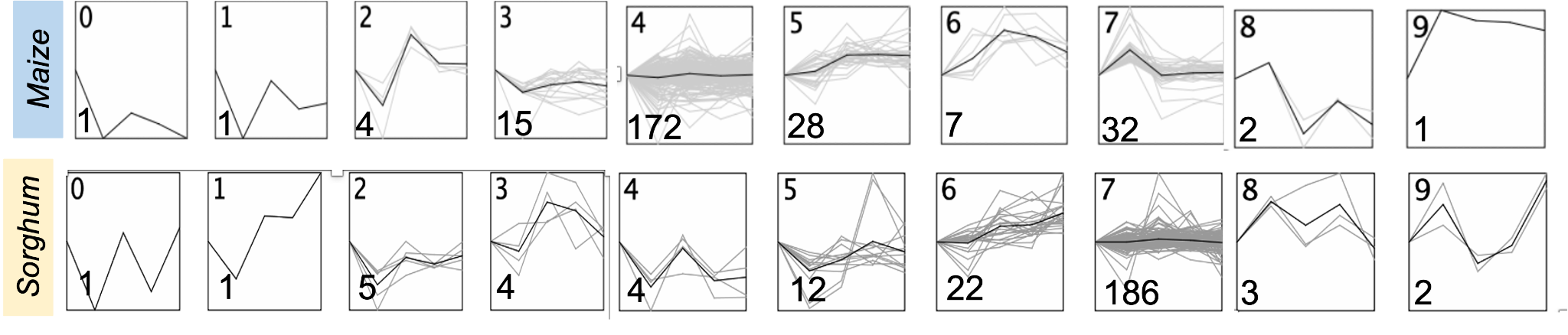


**Figure S9: Temporal transcriptomic dynamics in Maize and Sorghum under varying nitrogen conditions.** The expressed genes of the NUE GRN were categorized into ten clusters based on expression dynamics in leaf data. Gray lines represent individual gene expression trajectories, while black lines serve as the representative expression profile for each cluster.


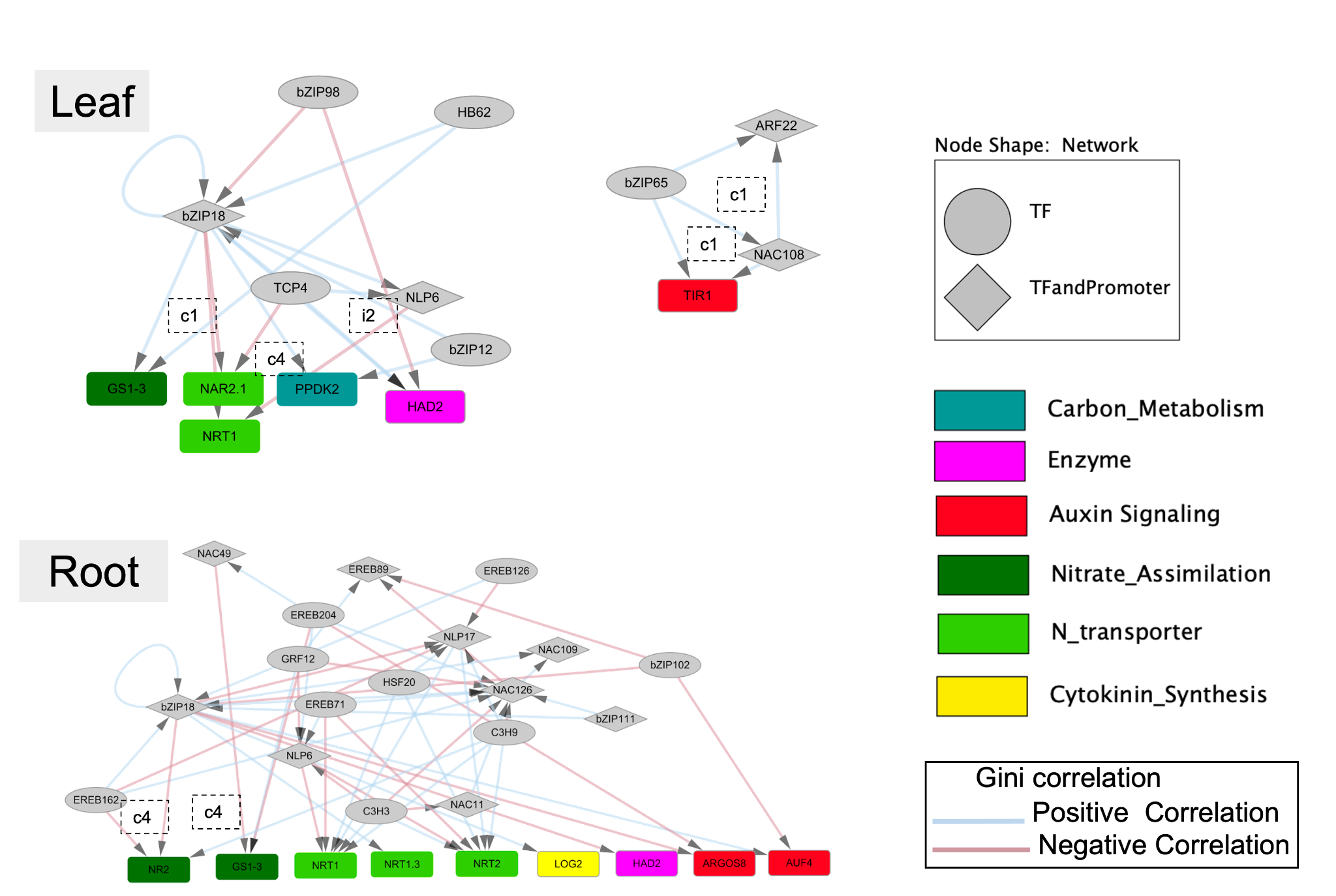


**Figure S10 Significant FFLs in Sorghum Leaf and Roots.** Positive Gini correlations are represented in blue, illustrating supportive interactions, and negative interactions are shown in pink, highlighting inverse relationships within the module. FFL loops: For coherent: c1 -Coherent Type 1, c2 -Coherent Type 2, c3 -Coherent Type 3, and c4 -Coherent Type 4 and for incoherent: c1 -Incoherent Type 1, c2 -Incoherent Type 2, c3 -Coherent Type 3, and c4 -Coherent Type 4.
